# Exosomal circ‐0100519 promotes breast cancer progression via inducing M2 macrophage polarisation by USP7/NRF2 axis

**DOI:** 10.1002/ctm2.1763

**Published:** 2024-08-06

**Authors:** Minyu Zhuang, Xiaoqiang Zhang, Jie Ji, Hongfei Zhang, Li Shen, Yanhui Zhu, Xiaoan Liu

**Affiliations:** ^1^ Breast Disease Center The First Affiliated Hospital of Nanjing Medical University Nanjing Jiangsu P.R. China; ^2^ Department of Breast Surgery Cancer Hospital of the University of Chinese Academy of Science (Zhejiang Cancer Hospital) Hangzhou China; ^3^ Department of Ultrasound in Medicine, Second Affiliated Hospital Zhejiang University School of Medicine Zhejiang China; ^4^ Department of General Surgery The Fourth Affiliated Hospital of Nanjing Medical University Nanjing Medical University Nanjing Jiangsu China

**Keywords:** breast cancer, circ‐0100519, exosomes, HIF‐1α, macrophages, NRF2, USP7

## Abstract

**Background:**

Breast cancer (BC) is one of the most prevalent malignant tumours that threatens women health worldwide. It has been reported that circular RNAs (circRNAs) play an important role in regulating tumour progression and tumour microenvironment (TME) remodelling.

**Methods:**

Differentially expression characteristics and immune correlations of circRNAs in BC were verified using high‐throughput sequencing and bioinformatic analysis. Exosomes were characterised by nanoparticle transmission electron microscopy and tracking analysis. The biological function of circ‐0100519 in BC development was demonstrated both in vitro and in vivo. Western blotting, RNA pull‐down, RNA immunoprecipitation, flow cytometry, and luciferase reporter were conducted to investigate the underlying mechanism.

**Results:**

Circ‐0100519 was significant abundant in BC tumour tissues and related to poor prognosis. It can be encapsulated into secreted exosomes, thereby promoting BC cell invasion and metastasis via inducing M2‐like macrophages polarisation.Mechanistically, circ‐0100519 acted as a scaffold to enhance the interaction between the deubiquitinating enzyme ubiquitin‐specific protease 7 (USP7) and nuclear factor‐like 2 (NRF2) in macrophages, inducing the USP7‐mediated deubiquitination of NRF2. Additionally, HIF‐1α could function as an upstream effector to enhance circ‐0100519 transcription.

**Conclusions:**

Our study revealed that exosomal circ‐0100519 is a potential biomarker for BC diagnosis and prognosis, and the HIF‐1α inhibitor PX‐478 may provide a therapeutic target for BC.

## Graphical abstract

1. HIF‐1α serves as an upstream effector to enhance circ‐0100519 transcription.

2. Exosomal circ‐0100519, secreted from cancer cells, can be engulfed by macrophages, thereby suppressing NRF2 ubiquitination via interacting with USP7 and NRF2.

3. The circ‐0100519/USP7/NRF2 axis promotes the progression of breast cancer through regulating M2 macrophage polarisation.

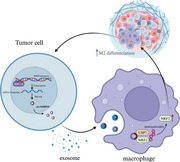

## 1 INTRODUCTION

Breast cancer (BC) is a greatly heterogeneous disease, which caused by a combination of factors. Based on the expression of oestrogen receptor (ER), progesterone receptor (PR) and human epithelial growth factor receptor 2 (HER2), BCs are clinically categorised into three major groups: ER^+^, HER2^+^ and triple negative breast cancer (TNBC).^1^ Even with the current therapeutic approaches, including comprehensive treatment based on surgery, a subset of breast cancer patients still confront the looming threat of postoperative recurrence, metastasis and chemotherapy resistance, posing a grave risk to patient survival.^2^ Thus, it is critical to further understand the molecular processes behind the advancement of BC to improve the prognosis of patients.

The tumour microenvironment (TME) is a complex ecosystem, composed of various cell types and secreted factors, which with both anti‐tumour and pro‐tumour effects.^3^ As the tumour progresses, it further utilises the immune and neuroendocrine systems to induce a homeostasis state which is conducive to expansion.^4^ Among the constituents of the TME, tumour‐associated macrophages (TAMs) are of paramount importance and extensively involved in modulating tumour progression.^5^ Depending on different microenvironments and stimuli, macrophages can polarise in various directions and primarily categorise into tumour‐suppressing (M1 phenotype) and tumour‐promoting (M2 phenotype) states.^6^, ^7^ The conspicuous plasticity and their crucial role in tumour advancement underscore that TAMs are poised to become pivotal targets in BC therapy.^8^

Exosomes are nano‐sized vesicles, with diameters ranging from 30 to 150 nm, secreted extracellularly by a variety of cells.^9^ Exosomes typically transfer a wide array of noncoding RNA, including circRNAs, microRNAs and long noncoding RNAs, and perform an essential part in the intricate network of intercellular communication.^10^, ^11^, ^12^ Mounting evidence has revealed that tumour‐derived exosomes facilitate tumourigenesis through various processes, such as promoting epithelial‐mesenchymal transition (EMT), inducing angiogenesis, mediating immune evasion and regulating macrophage polarisation.^13^, ^14^, ^15^ This study aimed to explore the mechanism by which exosomes regulate the TME of breast cancer.

CircRNAs represents a type of long noncoding RNAs characterised by a circular structure with covalent closure, devoid of a 5′ cap and 3′ poly(A) tail.^16^ Its circular conformation can resistant degradation by exonucleases and confer greater stability compared to linear RNAs. Numerous investigations have revealed that exosomal circRNAs play a critical role in the occurrence and progression of various tumours,^17^, ^18^ especially BC.^19^, ^20^, ^21^ For instance, Lin et al. explored that exosomal circPDK1 stimulated c‐myc activation to increase glycolysis in pancreatic cancer through the degradation of BIN1 and modulation of the miR‐628‐3p/BPTF axis.^22^ Lu et al. identified BC‐cell‐derived exosomes containing circ‐0001142 regulated macrophage polarisation via miR‐361‐3p/PIK3CB axis, which ultimately promoted BC progression.^23^ Nevertheless, the specifics processes by which circRNAs in exosomes regulate TAMs in BC still remains unknown. Gaining insights into the complex interactions that occur between BC and TAMs may thus lead to novel therapeutic approaches.

In this study, we identified circ‐0100519 upregulated in BC tumour tissues and closely related to macrophage infiltration through high‐throughput sequencing. In addition, circ‐0100519 was highly abundant in serum exosomes and positively correlated with poor prognosis. Tumour‐derived circ‐0100519 was delivered to macrophages by exosomes. Elevated circ‐0100519 promoted BC progression by shifting macrophage polarisation toward M2 phenotype. Mechanistically, circ‐0100519 enhanced the effect of USP7 on the deubiquitination and stability of NRF2 through acting as a scaffold in macrophages.

## 2 MATERIALS AND METHODS

### 2.1 Study patients

Patients from the Breast Center of the First Affiliated Hospital of Nanjing Medical University were identified. After removing individuals with underlying medical disorders and past cancer diagnoses, our research comprised 196 BC tissues for high‐throughput sequencing and 60 participants (BC tissues and matched adjacent tissues) for further real‐time quantitative reverse transcription PCR (qRT‐PCR) and in situ hybridisation (ISH) verification. Two board‐certified pathologists assessed the histopathology. The anonymised data on clinicopathological characteristics of 196 cases are included in Table S1. Table S2 contains the anonymised information on the clinicopathological features of 60 patients. The ethics committee of First Affiliated Hospital of Nanjing Medical University approved our study, and all patients provided informed consent.

### 2.2 Cell lines and main reagents

In this investigation, normal human breast epithelial cell lines (MCF‐10A), human BC cell lines (MDA‐MB‐231, MDA‐MB‐468, BT‐549, Hs578T, MCF‐7, SK‐BR‐3, T‐47D and ZR75‐1) and mouse BC cell line (4T1) were used. Subtypes of the above cell lines: Basal‐like: MDA‐MB‐231, MDA‐MB‐468, BT‐549, Hs578T, 4T1. Luminal: MCF‐7, T‐47D, ZR75‐1. HER2^+^: SK‐BR‐3. Mouse macrophages cell lines (BMDM) and human monocyte THP‐1 cells were applied. The mice tibia and femur bones were immersed in 75% ethanol upon their death. With a syringe, the bone marrow was removed and rinsed with PBS through a 70 µm filter. After gathering the flushing solution in a centrifuge tube, it was spun for 3 min at 1200 rpm. Then we toss the supernatant and added 1 mL red blood cell Lysis Buffer/ACK Lysis Buffer for 2 min. After centrifuging the tube for 5 more minutes at 1200 rpm, dispose of the supernatant. Cell precipitate was resuspended in DMEM with 20% fetal bovine serum (FBS) and 10% L929 cell supernatant, and grown in 5% CO_2_, 37°C incubator.

GW4869 (D1692, Sigma) was employed to prevent the generation of exosomes.

### 2.3 RNA sequencing

A total of 196 BC tumour tissues were collected for high‐throughput sequencing. The breast cancer tissues are taken out during surgery and kept in RNA later. Laboratory of Southeast University accomplished the task mentioned above. The illumina platform was used to sequence the libraries. Following the manufacturer instructions, total RNA (apart from ribosomal RNA, or rRNA) was isolated from tumour tissues. The extracted RNA was then promptly frozen in liquid nitrogen and kept at −80°C. RNase R was used to eliminate the linear RNAs. Ovation human FFPE RNA‐seq library systems (NuGEN Technologies, San Carlos, CA, USA) were used to create the RNA‐seq libraries, and the libraries were sequenced on the Illumina HiSeq X Ten platform (Illumina, San Diego, CA, USA). Normalised FPKM (fragments per kilobase of exon per million reads mapped) and count data were used to quantify the expression levels of genes. A total of 57 773 transcripts expression were measured.

### 2.4 Bioinformatic analyses

With the aid of Bioconductor packages ‘pheatmap’, ‘limma’, ‘DESeq2’, ‘corrplot’ and ‘ssGSEA’, we were able to identify the circRNA that showed the greatest association with the degree of macrophage infiltration. GSEA analysis was also used to conduct GO and KEGG pathway enrichment analysis for circ‐0100519 high and low expression. (|NES| > 1, *p* value < .05, FDR < 0.25) We used ‘survminer’ and ‘survival’ packages or GraphPad Prism version 8 (GraphPad Software) to perform survival analysis. Related date are included in Supplementary File 1, named ‘normalized_circRNA_expr.txt’ and ‘normalized_mRNA_expr.txt’.

### 2.5 Mice model assay

For the immune system investigation, 4T1 cells were injected into the mammary fat pad of 5‐week‐old female Balb/c mice via the nipple area. Following the injection of 5 × 10^4^/50 µL 4T1 cells (50 µL per mammary fat pad), Balb/c mice were given 50 µg exosomes in 50 µL PBS per mouse every 3 days for 4 weeks via tail vein injection (exosomes were isolated from supernatants of shNC‐ and shcirc‐0100519‐transfected BT‐549 cells).

For the downstream mechanism study, we gave each DTR mice (Vital River Laboratory Animal Technology) an intraperitoneal injection of 150 ng of diphtheria toxin (Sigma, D0564) to eradicate macrophages. Three days prior to the tumour creation until the tumour collection was the injection window. Following the injection of 5 × 10^4^/50 µL 4T1 cells (50 µL per mammary fat pad), 5‐week‐old female Balb/c mice were given 50 µg exosomes in 50ul PBS per mouse and 1 × 10^6^/50 µL BMDM‐NRF2 or BMDM‐Vector. (Exosomes were isolated from shNC‐ and shcirc‐0100519‐transfected BT‐549 cell supernatants. BMDMs were transfected with NRF2 or Vector.) Exosomes and BMDMs were injected into tail vein every 3 days for 4 weeks.

To further verify the role of NRF2, cmyeloid‐specific Cre mice (Gem Pharmatech) and NRF2‐LoxP mice (NRF2FL/FL) (Gem Pharmatech) mice were crossed to produce myeloid‐specific Nrf2‐deficient mice (also known as ‘NRF2^M‐KO^’). Female NRF2^M‐KO^ mice (5‐week‐old) on a Balb/c background were used in this study. NRF2 ^M‐KO^ mice were injected 5 × 10^4^/50 µL 4T1 cells into mammary fat pad via the nipple area and then given 50 µg exosomes in 50ul PBS per mouse every 3 days via tail vein injection (exosomes were isolated from shNC‐ and shcirc‐0100519‐transfected BT‐549 cells supernatants).

To verify the role of HIF‐1α serves as an upstream effector, 5‐week‐old female Balb/c mice were injected 4T1 cells transfected with circ‐0100519 into the mammary fat pad. Meanwhile, the Balb/c mice treated with HIF‐1α inhibitor PX‐478 (30 mg/kg body weight) or PBS via intraperitoneal injection twice a week.

All mice were euthanised via cervical dislocation after 4 weeks, and the tumour size was measured. Animal experimentation is ethically acceptable as 2019SR512.

### 2.6 Live bioluminescence imaging

For the lung metastasis model, 5‐week‐old female Balb/c mice received an injection of 5 × 10^4^/50 µL 4T1‐luc cells via the tail vein and followed by the treatment as described above. Mice were put to sleep using 2.5% isoflurane and given an intraperitoneal injection of D‐luciferin (Biosynth) at 150 µg/mL in PBS. The bioluminescence imaging system based on a charge‐coupled device camera was used for the bioluminescence imaging.

### 2.7 Cell and plasmids transfection

Circ‐0100519 overexpressed plasmid pcDNA3.1, circ‐0100519‐MUT and lentivirus pLV‐circ‐0100519 were purchased from Fenghui Biological Company. The ORFs clones were used to create constructs that generated Myc‐tagged USP7, Flag‐tagged NRF2 and HA‐UB (HEBIO, China) and inserted into either plasmids or lentivectors. A pCMV‐N‐Flag plasmid containing NRF2 segment was created and sequenced. The pCMV‐N‐Flag plasmid has been modified to accommodate the Flag‐NRF2 plasmid as well as the various NRF2 domains, such as NRF2‐Motif (29−82), NRF2‐Compositional bias (334−449), NRF2‐bZIP (497−560), and NRF2‐Region (571−605).

### 2.8 RNA extraction and qRT‐PCR assays

RNA Extraction Kit (Vazyme) was used to extract total RNA from cells and tissues. Utilising the reverse transcription technique (Vazyme), cDNA was created. Following that, SYBR Green PCR Master Mix was used for qRT‐PCR (Vazyme). The primers that were used are available in Table S3. To calibrate the original circ‐0100519 concentrations in cells, GAPDH expression was employed as a control. The 2^−ΔΔCT^ method was used to normalise the results of circ‐0100519 expression in different cells.

### 2.9 RNA immunoprecipitation (RIP)

As directed by the manufacturer, the Magna RIP RNA‐binding Protein Immunoprecipitation Kit (Millipore, USA) was used. Beads coated with IgG, anti‐USP7, or ant‐NRF2 antibodies (Millipore) were then treated with the cell lysates for an overnight period at 4°C on a plate. qRT‐PCR was used to identify the extracted RNA (using a RNeasy MinElute Cleanup Kit, Qiagen, USA).

### 2.10 RNA pull‐down assays

After we lysed the cells, THP‐1 cells were treated with a biotin‐labelled circ‐0100519 probe (RiboBio, China). Subsequently, cell lysates were treated at room temperature with agarose magnetic beads (Thermo Fisher Scientific, USA) coupled with streptavidin. Western blot and mass spectrometry were used to identify the interacting proteins.

### 2.11 Isolation, characterisation and quantitation of exosomes

Following the collection of the conditioned medium, the conditioned media were collected and centrifuged in the subsequent order: 300 × *g*, 15 min; 2000 × *g*, 15 min; 10 000 × *g*, 30 min; and 100 000 × *g*, 90 min at 4°C for ultracentrifugation (Beckman Coulter, Germany). Transmission Electron Microscope (TEM) and Nanoparticle Tracking Analysis (NTA) were used to examine the distribution of exosome sizes as well as their ultrastructure. In addition, the protein markers Calnexin, CD9 and TSG101 that are unique to exosomes were examined using Western blotting. Exosomes proteins were quantified by using BCA Protein Assay Kit (Beyotime, P0011) and utilise the quantified protein quantity to reflect the amounts of exosomes.

### 2.12 Coculture assay

Phorbol‐12‐myristate‐13‐acetate (PMA; Sigma–Aldrich, USA, 100 ng/mL) was used to induce the differentiation of THP‐1 cells into macrophages for 24 h and then the induced THP‐1 cells were cocultured with BT‐549 and T‐47D cells. BMDM was stimulated with 10 ng/mL IL‐4 (Peprotech) for 24 h and then cocultured with BT‐549 cells.

### 2.13 Cell proliferation assay

Cell viability was assessed using the CCK‐8 kit (Dojindo, Japan). Following a 48‐h treatment, 2 × 10^3^ BC cells were collected and subsequently planted onto 96‐well plates for further incubation. Following the directions of adding the detection reagent, we incubated the cells for 2 h at 37°C in the dark before measuring the absorbance at 450 nm. In order to assess BC cell proliferation capacity, 2 × 10^3^ cells were planted in 6‐well plates for the colony formation test. The treated cells were cultured for 24 h in 2 × 10^4^ cells per well in 24‐well plates. After fixation, membrane rupture, and staining (using EdU Assay Kit, RiboBio, China), the average number of cells in three sections of each sample was counted under a Leica microscope in order to determine the proliferation.

### 2.14 Transwell assay

A total of 2 × 10^4^ cells were planted in the upper layer of transwell membrane', and 10% fetal bovine serum was added to the lower chamber to encourage cell migration. Matrigel (BD Biosciences, MA, USA) was mixed after being diluted with 100 µL serum‐free media in the invasion assay. The cells that traversed the membrane were stained with crystal violet for 20 min and examined under a microscope following 24‐h incubation at 37°C in a 5% CO_2_ environment.

### 2.15 Flow cytometry

To determine the percentage of M2‐like macrophage both in vivo and in vitro, single‐cell suspension was cultured with dead‐FITC, CD45‐eFlour506, CD11b‐eFlour 450, F4/80‐PE, CD86‐PE‐Cy7 and CD206‐APC in accordance with the instructions. Beckman Coulter CytoFLEX flow cytometer was used to identify labelled cells. To analyse the data, FlowJo (Version 10.6.1) was utilised.

### 2.16 Western blot assay

The Radioimmunoprecipitation Assay (RIPA) Lysis Buffer (Beyotime) was used to lyse cells and tissues. After separating equivalent amounts of protein using SDS‐PAGE, the protein was moved to a membrane and incubated with corresponding primary and secondary antibodies. Image J software is used to process the outcomes.

### 2.17 Immunoprecipitation (IP) and immunoprecipitation coupled with mass spectrometry (IP/MS)

RIPA lysis solution (Thermo Scientific) was used to lyse BMDM and THP‐1 cells. Cellular proteins were incubated with primary antibodies at 4°C for duration of the night before addition of Protein A/G‐agarose beads (Beyotime). After being washed with lysis buffer 4 times, the precipitates were boiled in SDS sample buffer. Immunoblotting tests were performed on the resultant products. Mass spectrometry was also utilised for the extraction and analysis of the immunoprecipitations.

The extent of protein coverage and number of peptides identified in the mass spec experiments were listed in Table S4.

### 2.18 Deubiquitination assay

The designated plasmids were cotransfected into THP‐1 cells in order to perform the in vivo ubiquitination assay. The endogenous NRF2 ubiquitination is subsequently quantified by ubiquitin antibody immunoblotting. In addition, the exogenous ubiquitination of the NRF2 protein was assessed using a Co‐IP assay after the Flag‐NRF2 and Myc‐USP7 proteins were generated in vitro and exposed to a ubiquitin conjugation mixture.

### 2.19 Immunohistochemical staining (IHC), in situ hybridisation (ISH), RNA fluorescence in situ hybridisation (FISH) and TUNEL assay

In order to retrieve antigens, we cut up paraffin‐embedded tissues and then heated them to 95°C for 15 min. After that primary antibodies were incubated for a further night at 4°C. Sections were incubated at ambient temperature for 2 h after the application of HRP‐secondary antibodies. Haematoxylin and 3,3‐diaminobenzidine solutions were then used to stain the sections. After that, image J was utilised to evaluate and estimate the IHC staining density.

ISH test was used to identify circ‐0100519 on tissue microarrays of BC patients. Servicebio (Wuhan, China) designed the specific digoxin‐labelled probed of circ‐0100519.

Cy3‐labelled circ‐0100519 probes (RiboBio) were used to detect the subcellular location of circ‐0100519 in the FISH assay (emission at 570 nm).

The In Situ Cell Death Detection Kit (Servicebio, China) was utilised to perform the TUNEL experiment. The paraffin slices were processed according to protocol, including dewaxing, rehydrating and incubating. After three PBS washes, the samples were given the treatment of a TUNEL reaction mixture including TdT and dUTP (1:9) and then incubated for 2 h at 37°C in a humid atmosphere. The samples were counterstained with DAPI and seen under a fluorescence microscope. Apoptotic cells/total cells × 100% is the formula that was utilised to determine the cancer cells' apoptotic index.

### 2.20 Fluorescence microscopy

Using 4% paraformaldehyde, cell lines were frozen for 15 min. They were then cultured for 30 min using 0.5% Triton‐X, and lastly blocked for 30 min using 5% BSA. Primary antibody diluent was used and then incubated at 4°C for a whole night. After that, the samples were exposed to secondary antibodies for 2 h at room temperature. The cell nuclei were labelled with DAPI and photographs of colocalisation were evaluated using a Leica DMI3000 B confocal microscope.

### 2.21 CHIP assays

BT‐549 cells transfected with siCtrl/siHIF‐1α were cross‐linked at ambient temperature for 10 min using 1% formaldehyde. In brief, the specific steps include crosslinking and dissociation, ultrasound treatment, immunoprecipitation, elution and de‐crosslinking. The details were referred to the instructions of EZ CHIP KIT 22 ASSAYS (MILLIPORE; 17−371). qRT‐PCR was used to assay changes in HIF‐1α accumulation at the promoter region of circ‐0100519. ΔCt [normalised ChIP] = Ct [ChIP]—(Ct [Input] – Log2 (Factor of Input Dilution)); Factor of Input Dilution = (fraction of the input chromatin saved) – 1. %Input = 2(–ΔCt [normalised ChIP]) × 100%.

### 2.22 Statistics analysis

All statistical evaluations were completed using GraphPad Prism and R software (version 4.0.1). The values in bar graphs were shown as mean ± SD. We verified the normality of distribution and variance equality before beginning any parametric researches. An analysis of two groups was done using a two‐tailed unpaired Student's *t*‐test. One‐way ANOVA or two‐way ANOVA with Turkey's test were used to compare more than two groups (normality and equal variance passed). The groups with nonnormally distributed variables and/or unequal variance, the Wilcoxon rank‐sum test and the Kruskal–Wallis test were employed to analyse nonparametric data. The independent BC prognostic factors were assessed by cox proportional hazards model. Spearman correlation analysis was used to determine correlation coefficients. The survival curves were produced using the Kaplan–Meier approach, and the data were assessed using the log‐rank *t*‐test. ns, not significant; *p < .05, **p < .01, ***p < .001, ****p < .0001. The numbers of independent replicates and technical replicates in each group are listed in the figure legends. All the qRT‐PCR experiments set three technical replicates per biological replicate.

## 3 RESULTS

### 3.1 The identification of circ‐0100519 and its characteristics as a BC biomarker

**FIGURE 1:**
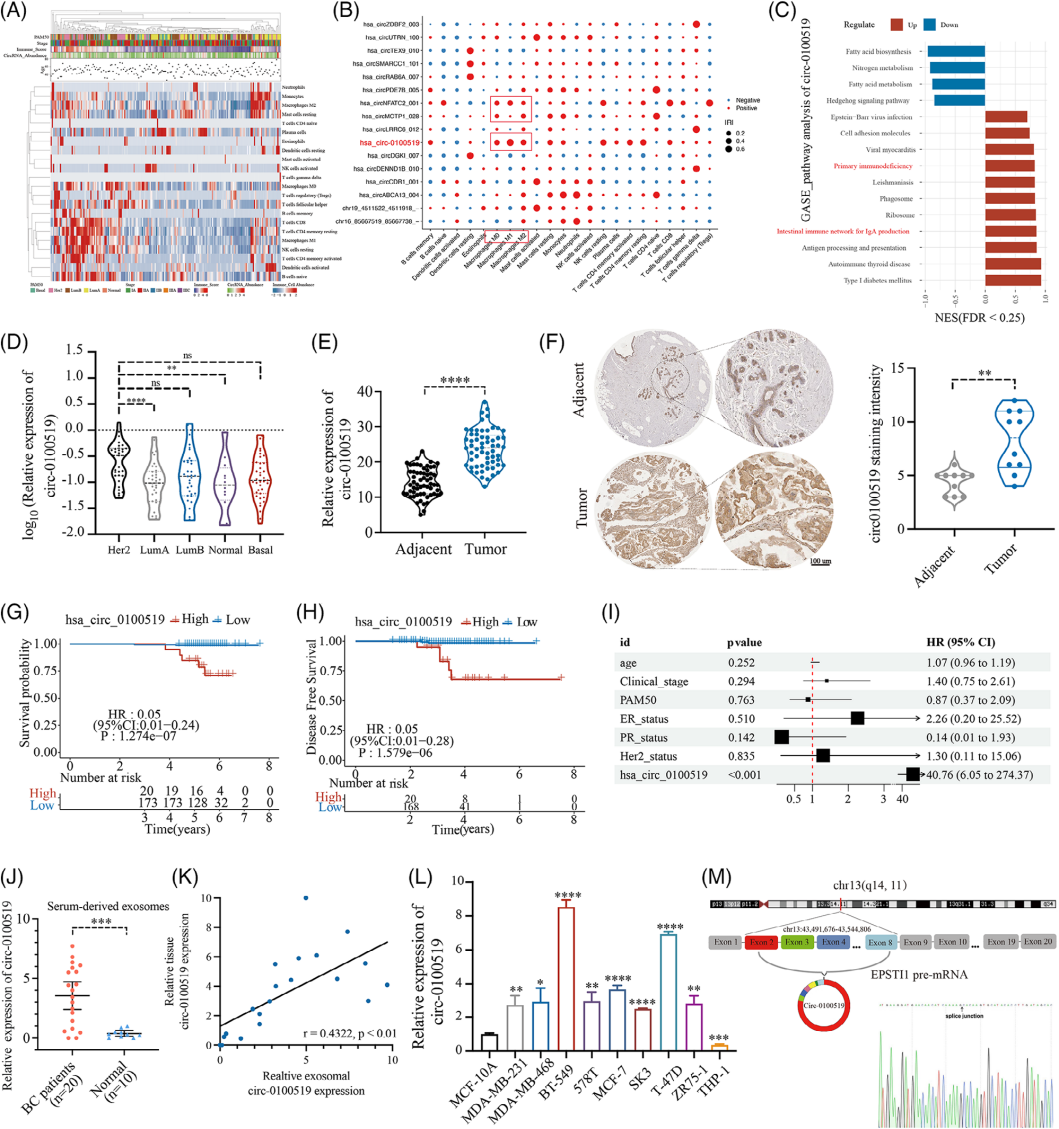
**The identification of circ‐0100519 and its characteristics as a BC biomarker**. (A) The heatmap for immune cell score displayed the distribution of immune score expression in several clinical characteristics (PAM50, stage, immune score, and age were shown), together with the abundance of circRNA. (B) Dot plot showing the connection between the top 16 circRNAs and several immune cells. The size of the dot reflected the significance of the correlation (blue represents negative; red represents positive). (C) The GASE pathway analysis of circ‐0100519. (D) The expression levels of circ‐0100519 in various PAM50 subtypes (*n* = 196 independent replicates). (E, F) The expression levels of circ‐0100519 in BC tumour tissues and matched adjacent tissues verified by qRT‐PCR (*n* = 60 independent replicates) and ISH (*n* = 10 independent replicates). Scale bar = 100 µm. (G, H) Overall survival and disease‐free survival of circ‐0100519 low and circ‐0100519 high groups in BC patients were analysed by Kaplan–Meier curves and log‐rank tests. (I) Risk factors associated with poor prognosis of 196 BC patients were assessed by multivariate cox regression analysis. (J) Circ‐0100519 in serum exosomes of normal people (*n* = 10 independent replicates) and BC patients (*n* = 20 independent replicates). (K) Correlation of circ‐0100519 expression between BC tumours and serum exosomes. (L) The expression levels of circ‐0100519 in MCF‐10A, BC cells and human monocyte THP‐1 cells were detected by qRT‐PCR. (M) The closed‐loop structure of circ‐0100519 was revealed using Sanger sequencing. A representative data set is displayed as mean ± SEM values. ns, not significant, *p < .05, **p < .01, ***p < .001, ****p < .0001.

To analyse the expression characteristics of circRNAs in BC, high‐throughput sequencing was used to detect circRNAs in tumour tissue samples from 196 patients. A total of 6073 circRNAs were identified (Figure S1A) and fraction of 22 immune cell types were analysed. Moreover, the association of immune cell types with different PAM50, clinical stages, immune scores, ages and circRNA abundance was shown in Figure 1A. Correlation between circRNAs and immune cell types was further demonstrated in general. A total of 16 circRNAs most strongly associated with each subtype of immune cells were selected (with statistical difference; |*R* ≥ 0.6|) (Figure S1B). As several studies have investigated that macrophage polarisation is a key regulatory factor of BC progression, bioinformatic analysis found that circNFATC2_001, circMCTP1_028 and circ‐0100519 expression were significantly related to the infiltration of macrophages (Figure 1B).^24^ We selected these three circ‐RNAs to be knocked down separately in tumour cells and cocultured with preactivated THP‐1 cells. Flow cytometry suggested that only circ‐0100519 can affect macrophage polarisation levels (Figure S1C–E). We selected circ‐0100519 for further Gene Set Enrichment Analysis (GSEA) and found that circ‐0100519 was more linked with primary immunodeficiency and intestinal immune network for IgA production (Figure 1C). Thus, we concentrated on circ‐0100519 and searched for the mechanism via which it modifies the polarisation of macrophages and controls the progression of BC. Based on RNA sequencing, Figure 1D depicted the expression levels of circ‐0100519 in various PAM50 subtypes. We further gathered 60 samples from BC tumour and matched adjacent tissues. Using real‐time quantitative reverse transcription PCR (qRT‐PCR) and in situ hybridisation (ISH), it was confirmed that tumour tissues had considerably elevated levels of circ‐0100519 (Figures 1E and F and S3J). Furthermore, Kaplan–Meier survival curve and multivariate cox regression established circ‐0100519 as an independent factor leading to poor BC prognosis (Figures 1G‐1I and S1F). CircRNAs have been commonly found in serum exosomes and may serve as a promising tumour biomarker.^25^, ^26^ In order to investigate whether circ‐0100519 could be found in serum exosomes and its capacity as a tumour biomarker, we took blood samples from twenty patients with BC and 10 healthy individuals as controls. Circ‐0100519 derived from BC patients' serum exosomes showed abundantly expression, while it hardly expressed in the serum exosomes of healthy individuals (Figure 1J). In addition, the expression levels of circ‐0100519 in serum exosomes were in line with those in matched BC tumours (Figure 1K). Taken together, these data suggested that circ‐0100519 is markedly upregulated in BC tumour tissues and serum exosomes, which make it possible to be a potential biomarker for early BC diagnosis. We further examined circ‐0100519 expression in normal mammary epithelial, BC and human monocyte THP‐1 cells and found that BC cells contained higher levels of circ‐0100519. For this investigation, the two cells (BT‐549 and T‐47D) exhibiting the greatest levels of expression were chosen (Figure 1L). Circ‐0100519 was generated from exons 2−8 of EPSTI1; the closed‐loop structure of circ‐0100519 was revealed using sanger sequencing (Figure 1M). In addition, we created convergent and divergent primers to amplify circ‐0100519 and its linear version. Circ‐0100519 could only be amplified by the agarose gel electrophoresis analysis, from cDNA rather than gDNA (Figure S1G). Treatment with RNase R or Actinomycin D showed that circ‐0100519 was stabilised in BC cells in comparison to linear EPSTI1 mRNA (Figure S1H and I). The findings of subcellular fractionation and FISH experiment indicated that circ‐0100519 was mostly localised in the cytoplasm (Figure S1J and K). All together, these results showed that circ‐0100519 is an abundant and stably expressed cytoplasmic circRNA in BC.

### 3.2 Exosomal circ‐0100519 promotes BC cell proliferation and metastasis in vitro

**FIGURE 2:**
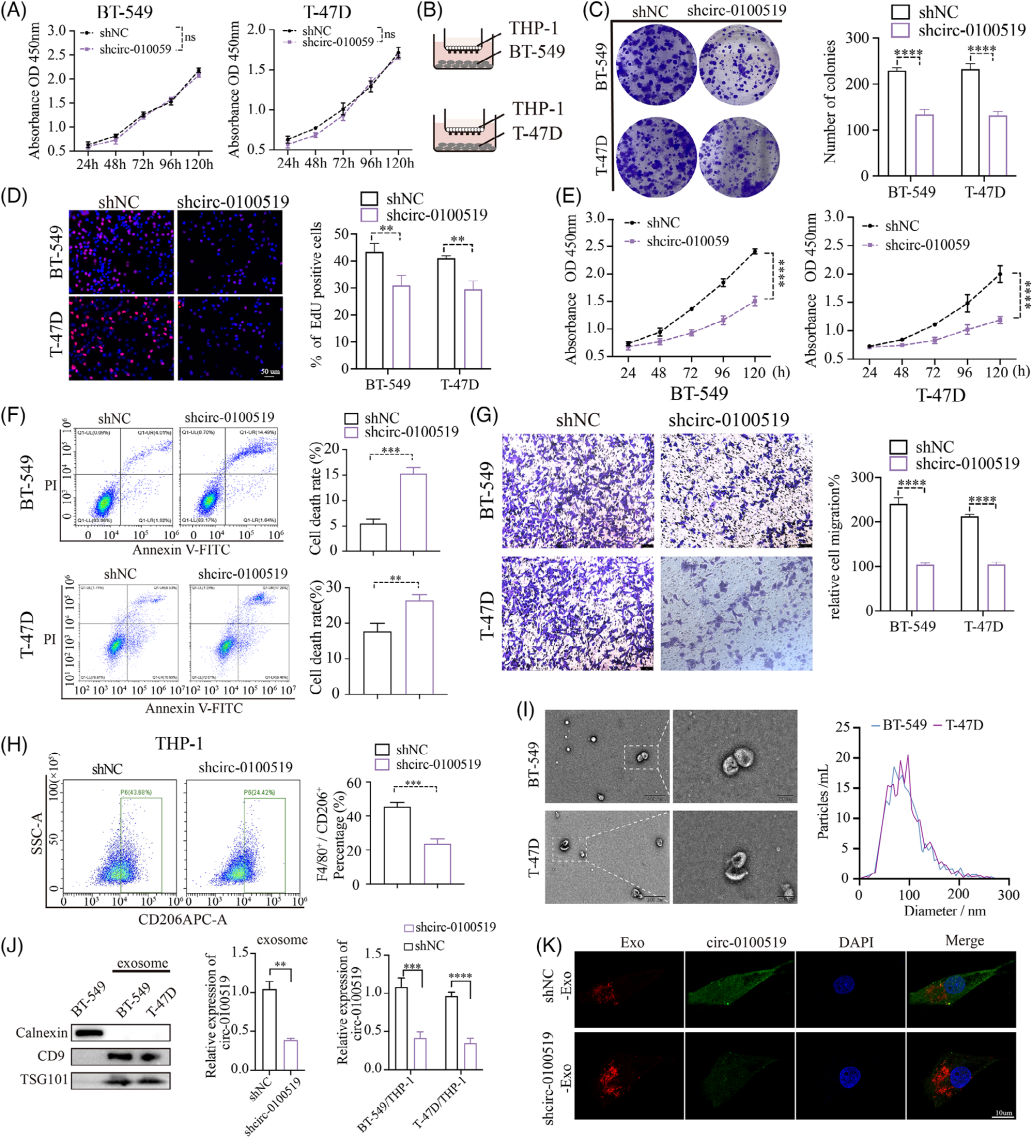
**Exosomal circ‐0100519 promotes BC cell proliferation and metastasis in vitro**. (A) CCK‐8 assays were used to assess the viabilities of BC cells after treated with shNC or shcirc‐0100519. (B) Schematic diagram showing preactivated THP‐1 cocultured with BT‐549 or T‐47D. (C, D) Colony formation and EdU assays (scale bar = 50 µm) were used to detect the proliferative potential of BC cells. (E) The viabilities of BC cells were showed by CCK‐8 assays. (F) Analysis of the effect of downregulated circ‐0100519 expression on BC cell apoptosis. (G) Transwell migration assays were used to identify migration ability of BC cells. Scale bar = 1 mm. (H) FCM was used to assess CD206 expression in THP‐1. (C–H: BT‐549 or T‐47D cells were treated with shNC or shcirc‐0100519 before coculturing with THP‐1.) (I) TEM and NTA were used to identify exosomes obtained from the BT‐549 and T‐47D cell supernatants. The peak diameters of BT‐549‐Exo and T‐47D‐Exo were 80 and 95 nm, respectively. Scale bar = 50 nm. (J) Exosomes’ biomarker analysis using Western blot. The circ‐0100519 expression levels in exosomes obtained from the BT‐549 and T‐47D cell supernatants. The circ‐0100519 expression levels in macrophages treated with indicated treatments. (K) Confocal microscopy was used to verify macrophages can engulf exosomes and circ‐0100519. Scale bar = 10 µm. A representative data set is displayed as mean ± SEM values of three independent replicates. ns, not significant, *p < .05, **p < .01, ***p < .001, ****p < .0001.

We transfected shcirc‐0100519 in BT‐549 cells and found that EPSTI1 levels were not affected, while expression of circ‐0100519 were decreased which indicated that circ‐0100519 was a posttranscriptional level change (Figure S2A). To explore the role of circ‐0100519 in BC, we silenced circ‐0100519 in BT‐549 and T‐47D cells and found that downregulation of circ‐0100519 did not inhibit tumour cell proliferation in vitro (Figure 2A). Since TAMs are potent inducers of tumourigenesis and circ‐0100519 was closely associated with macrophage infiltration founded by our previous bioinformatic analysis, we speculated that the functional circ‐0100519 involved in BC through macrophages. Therefore, we cocultured BT‐549 and T‐47D cells with pretreated THP‐1 cells, colony formation, EdU assays, CCK‐8 assays, flow cytometry of cell apoptosis, and transwell assays were used to investigate the effects of circ‐0100519 on BC cells proliferation and metastasis in vitro (phorbol‐12‐myristate‐13‐acetate (PMA) was employed to induce the differentiation of THP‐1 cells into macrophages). The results demonstrated that downregulation of circ‐0100519 notably suppressed cell viability and migration (Figure 2B–G). Flow cytometry suggested that the proportion of F4/80^+^CD206^+^ cells was noticeably lower in the shcirc‐0100519 group than that in the shNC group (Figure 2H). Additionally, we further confirmed the flow cytometry results using BMDM (Figure S2B and C). By detecting the release of M1‐like cytokines and chemokines by THP‐1 coculture with BT‐549, we discovered that circ‐0100519 failed to affect M1 activation of TAMs, indicating the inhibition of circ‐0100519 only impacted M2 activation (Figure S2D). Exosomes are essential for the communication between BC cells and TAMs.^27^ The exosomes obtained from the supernatants of BT‐549 and T‐47D cells were assessed by TEM and NTA, which displayed typical circular particles. The peak diameters of BT‐549‐Exo (BT‐549‐Exosomes) and T‐47D‐Exo were 80 and 95 nm, respectively (Figure 2I). Western blotting showed that the exosomes we isolated expressed the same exosome surface indicators, such as CD9 and TSG101, but lacked the marker Calnexin. We next assessed the amount of circ‐0100519 in exosomes and found that circ‐0100519 was impoverished in exosomes when we downregulated circ‐0100519 in tumour cells. To explore whether exosomes containing circ0100519 can transport to macrophages, we cocultured BC cells with PMA‐induced THP‐1 cells and calculated the content of circ‐0100519 in macrophages by qRT‐PCR (Figure 2J). To further verify that macrophages can engulf exosomes and circ‐0100519, we cocultured exosomes with macrophages and observed that the macrophages internalised the red fluorescence‐labelled exosomes as well as exosome‐encapsulated circ‐0100519 labelled by green fluorescence, as evaluated by confocal microscopy (Figure 2K). In addition, when we treated the coculture system with the exosome inhibitor GW4869, the tumour‐promoting effects of circ‐0100519 shown above were reversed (Figure S2E). Instead of coculture system, GW4869 or DMSO was directly applied to breast cancer cells, no significant difference in cell proliferation was found (Figure S2F). Collectively, these data suggested that circ‐0100519 can be encapsulated and released by exosomes. There might be no exosome‐independent effects involved in this study.

### 3.3 Exosomal circ‐0100519 promotes BC development via altering the polarisation of M2‐like macrophages in vivo

**FIGURE 3:**
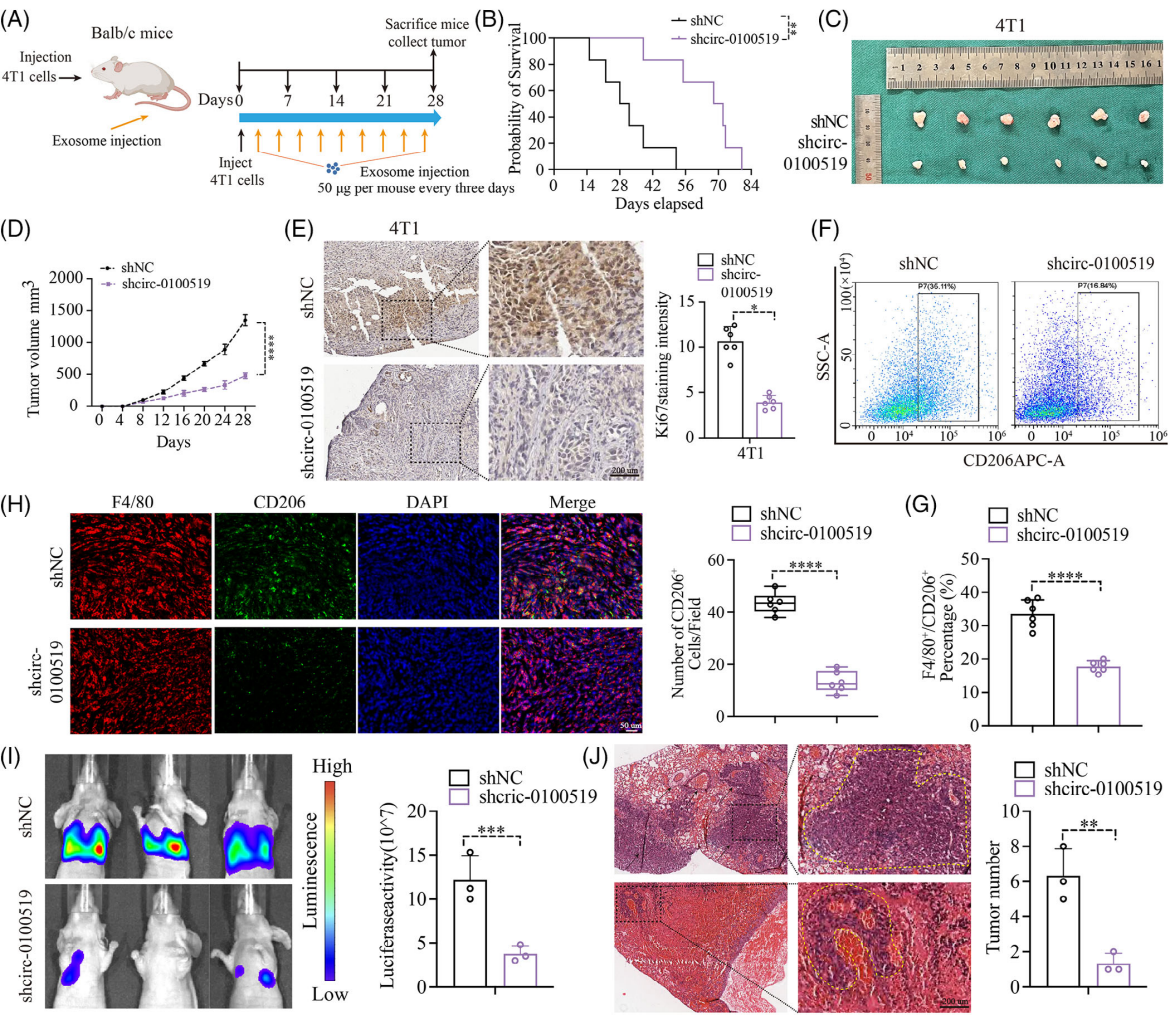
**Exosomal circ‐0100519 promotes BC development via altering the polarisation of M2‐like macrophages in vivo**. (A) The mouse tumourigenicity model's workflow. (B) The Kaplan–Meier curve was provided. (C) Photographs of tumours collected from 4T1‐bearing Balb/c mice (*n* = 6 per group). (D) The curve graph exhibited the tumour volume measured at different time‐points (*n* = 6 per group). (E) Ki67 IHC staining was applied to tumour sections (*n* = 6 for each group). Scale bar = 200 µm. (F, G) FCM was utilised to evaluate CD206 expression in Balb/c mouse tumours. (H) Immunofluorescent images of mouse tumour sections using F4/80 and CD206 antibodies. Scale bar = 50 µm. (I) Bioluminescence images of lung metastasis model injected with shNC or shcirc‐0100519 exosomes via tail vein injection. (J) Lung metastatic nodules were showed by H&E staining. Scale bar = 200 µm. A representative data set is displayed as mean ± SEM values of three to six independent replicates. ns, not significant, *p < .05, **p < .01, ***p < .001, ****p < .0001.

We cocultured THP‐1 cells with gradient concentrations of exosomes isolated from BT‐549 cells (0, 10, 25, 50, 100 µg) to evaluate the expression of circ‐0100519 in macrophages. We chose 50 µg as the optimal concentration for the subsequent experiments based the qRT‐PCR results (Figure S2G). To validate the carcinogenic effects of exosomal circ‐0100519 in vivo, a mouse tumourigenicity model (4T1 cell line) was constituted and intravenously injected with BT‐549 generated exosomes (exosomes were isolated from shNC‐ or shcirc‐0100519‐transfected cells supernatants) 50 µg each mouse every third day. The mice were sacrificed on day 28 with the tumours collected. As expected, injections of shcirc‐0100519 exosomes prolonged the survival time, and decreased the volume and weight of tumours, compared to the control group (Figure 3A–D). Also, the Ki67 proliferation index was reduced in the tumour‐bearing mice treated with shcirc‐0100519 exosomes (Figure 3E). To explore how shcirc‐0100519 exosomes suppressed tumour expansion, we looked into whether these shcirc‐0100519 exosomes‐induced malignancies showed polarisation‐suppressed M2 macrophage activity. In contrast to the shNC exosomes group, M2 marker expression was decreased after shcirc‐0100519 exosomes treatment (Figure 3F and G). Additionally, the tumour tissues of mice given shcirc‐0100519 exosomes treatment expressed fewer F4/80^+^CD206^+^ macrophages (Figure 3H). Using a lung metastasis model, we discovered that the tumour burden and incidences of lung metastasis in the shcirc‐0100519 group was lower than the shNC group (Figure 3I and J). Moreover, we collected pathological puncture samples from five BC patients (initial diagnosed in the fourth clinical stage) and found that the expression levels of circ‐0100519 in tumour tissues of patients with metastatic breast cancer were significantly higher than those of nonmetastatic patients which was in consistent with the lung metastasis mice model (Figure S2H). These results align with the in vitro data suggesting that the exosomal circ‐0100519 could also promote BC tumour proliferation and metastasis in vivo via encouraging M2‐like macrophages polarisation.

### 3.4 circ‐0100519 suppresses NRF2 ubiquitination via interacting with USP7 and NRF2

**FIGURE 4:**
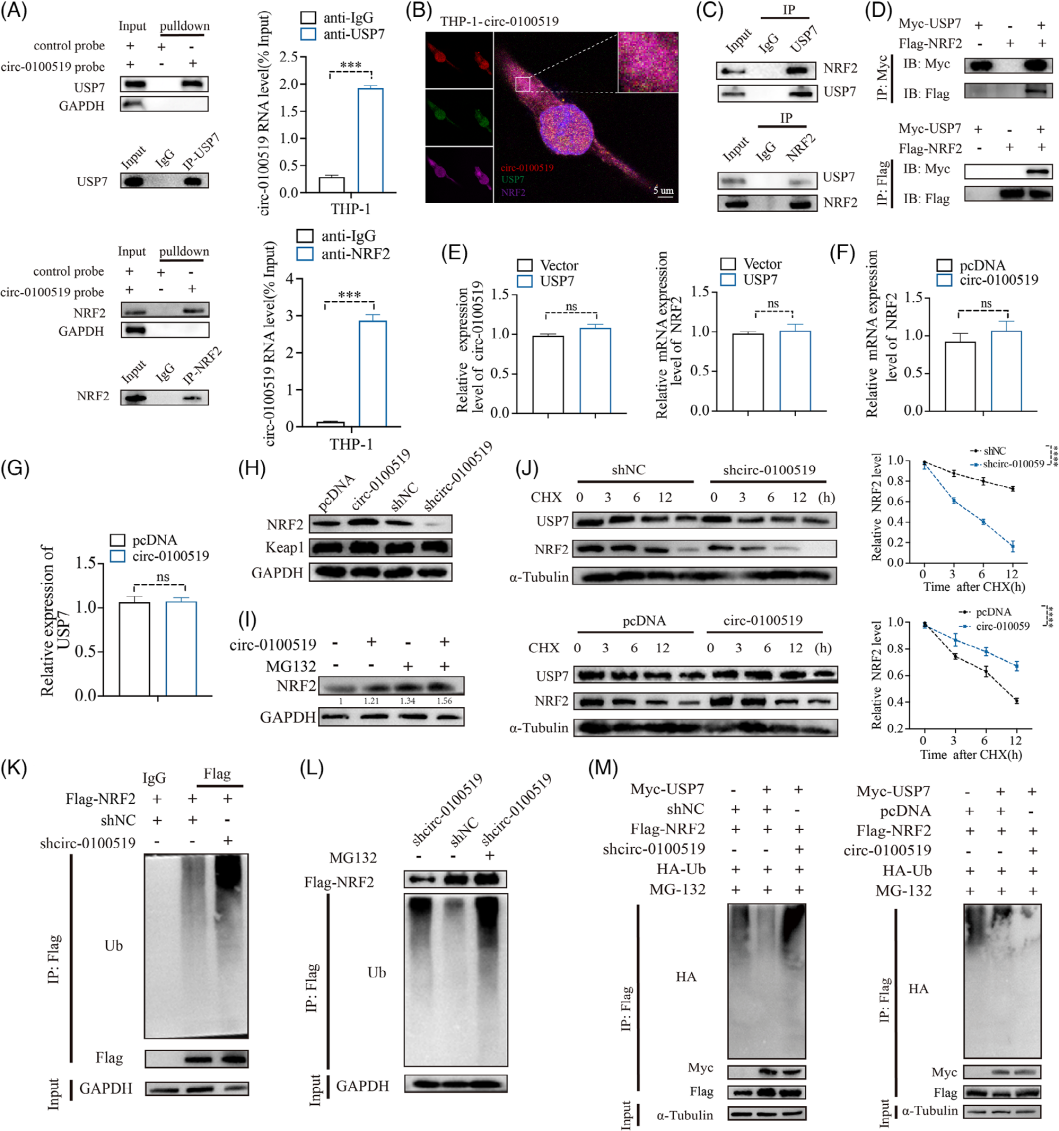
**circ‐0100519 suppresses NRF2 ubiquitination via interacting with USP7 and NRF2**. (A) RNA pull‐down employed the biotinylated circ‐0100519 probe to identify the interaction of circ‐0100519 with USP7 or NRF2 in macrophage. RIP assay further confirmed the interaction between circ‐0100519 and USP7/NRF2 with USP7 antibody/NRF2 antibody. (B) Colocalisation of circ‐0100519 (RED) with USP7 proteins (GREEN) and NRF2 proteins (PURPLE). Scale bar = 5 µm. (C) Co‐IP assays verified the binding association of circ‐0100519 with USP7 and NRF2 in THP‐1 cells. (D) Exogenous protein interactions were identified in THP‐1 cells. (E) Expression of circ‐0100519 and NRF2 RNA levels in THP‐1 transfected with USP7 or Vector. (F, G) Expression of NRF2 and USP7 RNA levels in THP‐1 transfected with circ‐0100519 or pcDNA. (H) The expression of NRF2 and Keap1 in protein level after overexpression or knockdown of circ‐0100519 in THP‐1. (I) The NRF2 protein level in THP‐1 with circ‐0100519 overexpression (using proteasome inhibitor MG132). (J) The USP7 and NRF2 protein level in specified time point following treatment with cycloheximide (CHX, 10 µg/mL) in transfected THP‐1 cells. (K) IP assays showed the ubiquitination modification level of NRF2 in THP‐1 cells with indicated treatments. (L) NRF2 protein and ubiquitination levels in THP‐1 transfected with shcirc‐0100519 or shNC were analysed using Western blot, either in the presence or absence of the proteasome inhibitor MG132. (M) Ubiquitination modification levels of NRF2 in THP‐1 cells after circ‐0100519 overexpression or knockdown. A representative data set is displayed as mean ± SEM values of three independent replicates. ns, not significant, *p < .05, **p < .01, ***p < .001, ****p < .0001.

We investigated whether circ‐0100519 performed its functions through interaction with RNA‐binding proteins using immunoprecipitation (IP) and LC‐MS/MS screening. We found that USP7 and NRF2 were included in the top 10 enriched proteins (Table S4). As several studies have documented that NRF2 is a key transcription factor that drives M2‐like macrophages polarisation and USP7 regulates multiple protein levels through deubiquitination.^28^, ^29^, ^30^ Therefore, it made us to assume USP7 and NRF2 might be the most likely RNA‐binding proteins of circ‐0100519 in THP‐1 cells (Figure S3A and B). Subsequently, RNA pull‐down and RIP assays verified this hypothesis, while the control circRNA did not bind with USP7 and NRF2 (Figure 4A). Moreover, IF and FISH experiments demonstrated that circ‐0100519 was colocalised with USP7 and NRF2 in the cytoplasm (Figures 4B and S3C). To determine whether NRF2 interacts with USP7 in macrophages, endogenous and exogenous coimmunoprecipitation (Co‐ IP) assay was performed, which showed that USP7 bound to NRF2 (Figures 4C and D and S3D). The qRT‐PCR results showed that USP7 had no effect on the RNA levels of NRF2 and circ‐0100519. Similarly, the NRF2 protein levels were dramatically regulated after circ‐0100519 was altered, with no effect on NRF2 mRNA levels, USP7 mRNA levels and USP7 protein levels, suggesting that circ‐0100519 may regulate NRF2 at the protein translation level through USP7 rather than the transcript level. Andrew et al. explored that USP7 is crucial to the development of tumours as it regulates P53 via MDM2.^31^ However, changes of circ‐0100519 levels did not impact the protein levels of P53 and MDM2 in our study. Also, the Keap1‐NRF2 pathway in a very well‐known oxidative stress pathway.^32^, ^33^ We speculated whether Keap1 played a role in circ‐0100519 promoting breast cancer progression. Notably, we discovered that Keap1 protein levels were not affected after circ‐0100519 altered (Figures 4E–H and S3E and F). These data indicated that the USP7/MDM2/p53 axis and the Keap1‐NRF2 pathway were not involved in the downstream regulation of circ‐0100519. In addition, proteasome inhibitors (MG132) could restore the elevated NRF2 protein levels caused by circ‐0100519 expression (Figure 4I). We also examined how the protein synthesis inhibitor cycloheximide (CHX) affected the stability of the endogenous NRF2 protein level in response to circ‐0100519 up‐ or down‐regulation. Overexpression of circ‐0100519 markedly inhibited the degradation of NRF2, whereas knockdown of circ‐0100519 significantly promoted NRF2 degradation (Figure 4J). Suppression of circ‐0100519 significantly enhanced the ubiquitination levels of NRF2 (Figure 4K). Also, the proteasome inhibitor MG132 treatment restored the shcirc‐0100519‐induced decrease in NRF2 protein levels, which implied that NRF2 protein levels were regulated through the ubiquitination‐proteasome pathway (Figure 4L). Next, we clarified whether circ‐0100519 was necessary for the deubiquitinating enzyme activity of USP7 on NRF2. The findings showed that the impact of USP7 on the deubiquitination and stability of NRF2 was significantly weakened by the loss of circ‐0100519 (Figure 4M). Collectively, circ‐0100519 may act as a scaffold to improve the binding of USP7 and NRF2, which could facilitate the effects of USP7 on NRF2.

**FIGURE 5:**
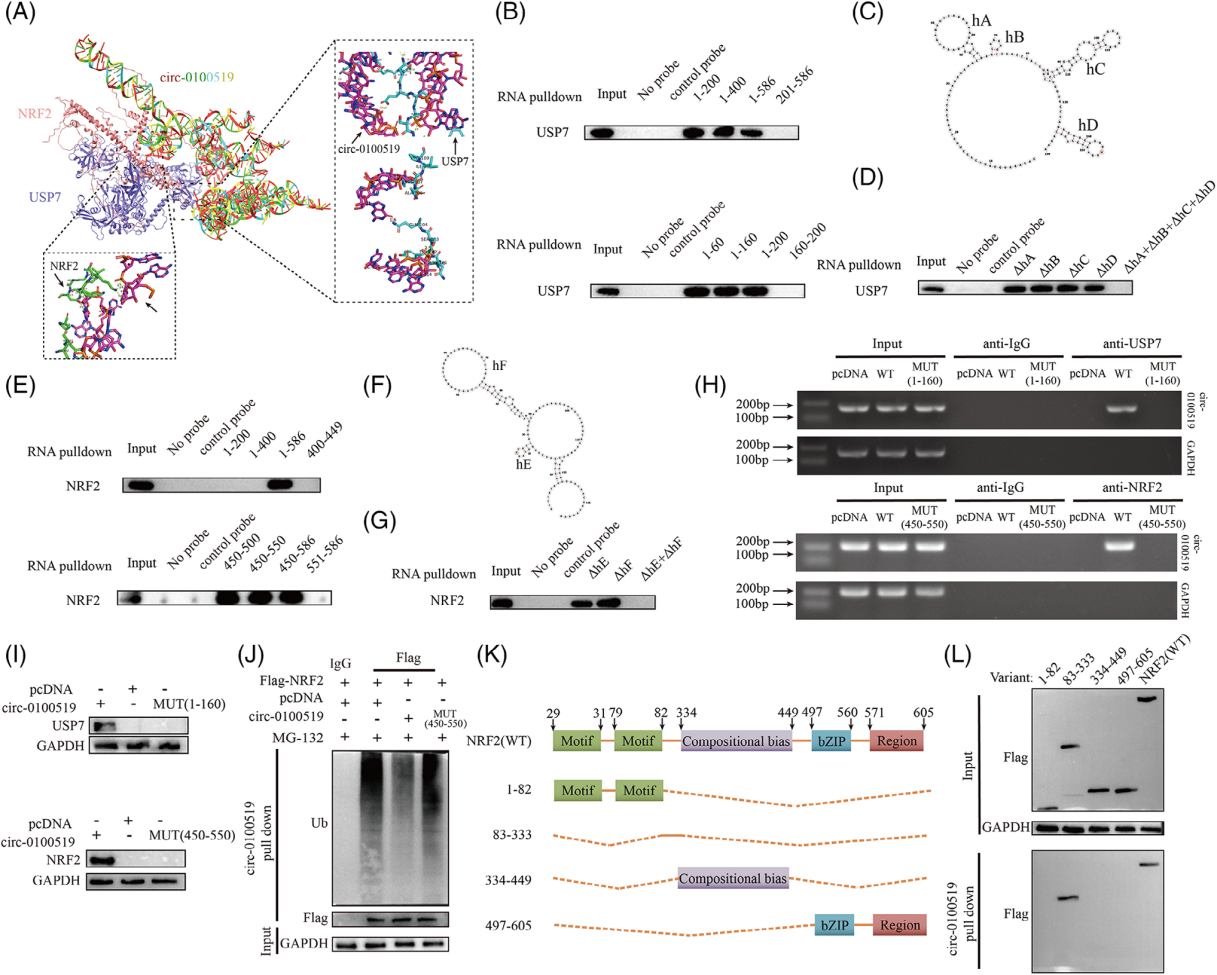
**circ‐0100519 suppresses NRF2 ubiquitination via interacting with USP7 and NRF2**. (A) 3D structure was employed to predict the precise binding positions for the interaction of circ‐0100519, USP7 and NRF2. (B) RNA pull‐down employing sequentially deleted circ‐0100519 fragments showed the binding region of circ‐0100519 with USP7. (C) The structure of circ‐0100519 fragment (nt 1−160) predicted by RNAfold. (D) Deletion of hA, hB, hC and hD (ΔhA+ΔhB+ΔhC+ΔhD) abolished the binding of circ‐0100519 fragment (nt 1−160) with USP7. (E) RNA pull‐down employing sequentially deleted circ‐0100519 fragments showed the binding region of circ‐0100519 with NRF2. (F) The structure of circ‐0100519 fragment (nt 450−550) predicted by RNAfold. (G) Double deletion of hE and hF (ΔhE + ΔhF) abolished the binding of circ‐0100519 fragment (nt 450−550) with NRF2. (H) RIP assays were performed employing anti‐USP7 or anti‐NRF2 antibodies in THP‐1 cells by agarose gel electrophoresis analysis. (I) RNA pull‐down assays were used to verify the interaction between circ‐0100519‐WT or circ‐0100519‐MUT and USP7/NRF2. (J) IP assays showed the ubiquitination modification level of NRF2 in THP‐1 cells with indicated treatments. (K) Schematic illustration of NRF2, displaying the wild‐type and truncations of NRF2. (L) The truncated NRF2 mutant harbouring 83−333aa retained the binding ability with circ‐0100519. Full length or the NRF2 fragments was transfected into THP‐1 cells for circ‐0100519 RNA‐pull down assay. A representative data set is displayed as mean ± SEM values of three independent replicates. ns, not significant, *p < .05, **p < .01, ***p < .001, ****p < .0001.

**FIGURE 6:**
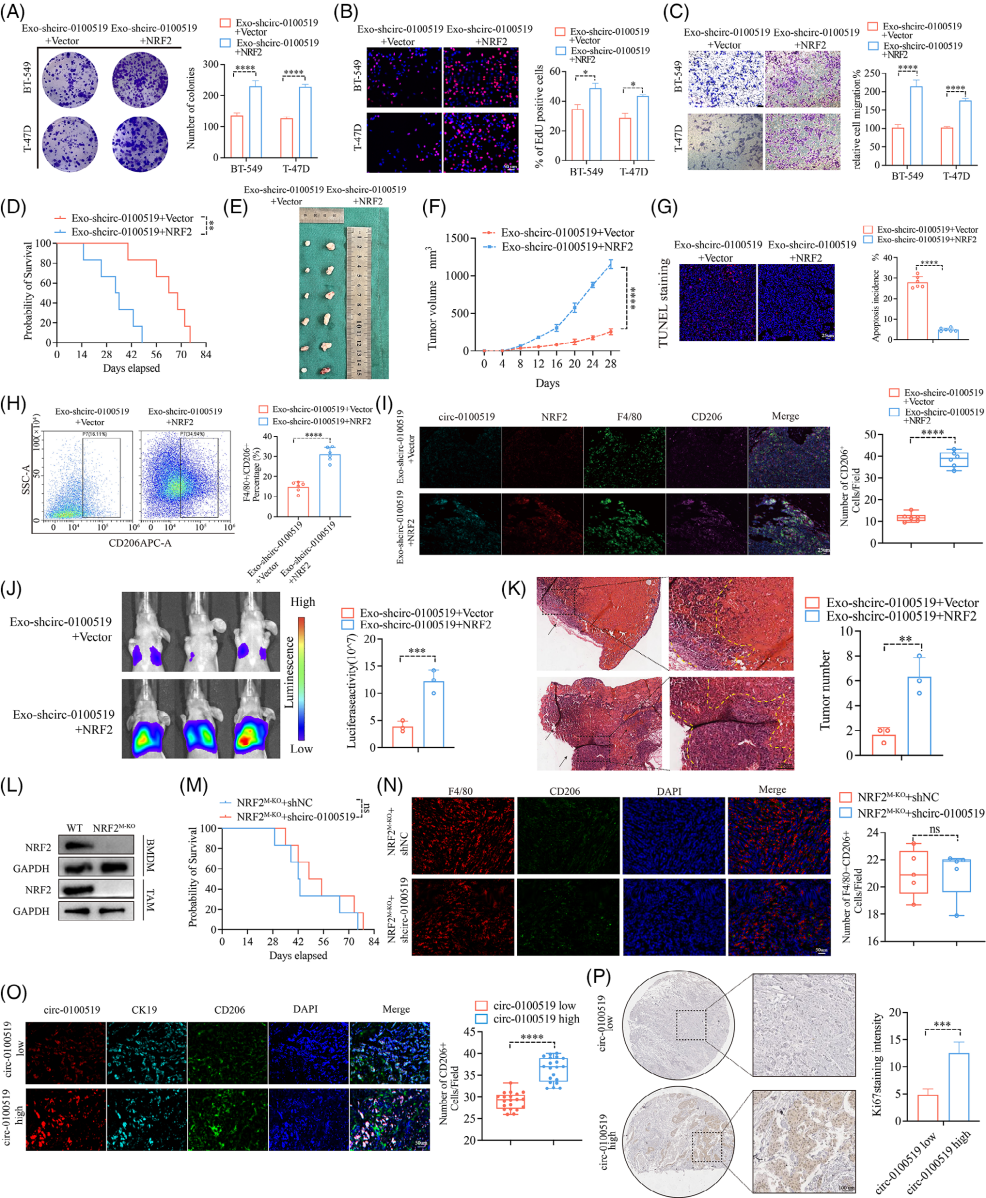
**NRF2 is a functional downstream mediator of circ‐0100519 and USP7**. (A–C) Colony formation, EdU assays (scale bar = 50 µm), and Transwell migration assays (scale bar = 1 mm) were used to detect the proliferative and migration potential of BC cells. (THP‐1 cells transfected with Vector or NRF2 plasmids were cocultured with exosomes isolated from circ‐0100519 knockdown tumour cells.) (D) The Kaplan–Meier curve was provided. (E) Photographs of tumours collected from 4T1‐bearing Balb/c mice with indicated treatment (*n* = 6 per group) (F) The curve graph exhibited the tumour volume measured at different time‐points (*n* = 6 per group). (G) Tumour sections were stained with TUNEL. (H) FCM was utilised to evaluate CD206 expression in Balb/c mouse tumours. (I) Immunofluorescent images of mouse tumour sections using circ‐0100519, NRF2, F4/80 and CD206 antibodies. Scale bar = 25 µm. (J) Bioluminescence images of lung metastasis model with indicated treatments. (K) Lung metastatic nodules were showed by H&E staining. Scale bar = 200 µm. (L) Western blotting showed NRF2 protein levels in BMDMs or TAMs from WT and NRF2^M‐KO^ mice. (M) Kaplan–Meier overall survival curves of NRF2^M‐KO^+shNC and NRF2^M‐KO^+shcirc‐0100519 mice with indicated treatment. (N) Immunofluorescent images of mouse tumour sections using F4/80 and CD206 antibodies. Scale bar = 50 µm. (O) Multicolour immunofluorescence using circ‐0100519, CK19, and CD206 antibodies in patient tumour sections (*n* = 20 pairs). Scale bar = 50 µm. (P) Patient tumour sections were stained with Ki67 IHC. Scale bar = 100 µm. A representative data set is displayed as mean ± SEM values of three to six independent replicates. ns, not significant, *p < .05, **p < .01, ***p < .001, ****p < .0001.

To elucidate how circ‐0100519 interacts with USP7 and NRF2, a series of circ‐0100519 mutants were created to identify the binding motifs of circ‐0100519 with USP7 and NRF2. By using RNA pull‐down assay, it was shown that whereas other mutants entirely lost their ability to bind to USP7, circ‐0100519 mutants with nt 1−160 retained this ability (Figure 5A and B). We next employed RNAfold to forecast the secondary structure of circ‐0100519 (nt 1−160) and identified four hairpins within nt 1−160, which we named hairpin A (hA), hairpin B (hB), hairpin C (hC) and hairpin D (hD) (Figure 5C). To further assess the contribution of four hairpins to USP7 binding, another set of nt 1−160 fragments was utilised, in which every hairpin was eliminated separately. The fragment that removes hairpins (ΔhA + ΔhB + ΔhC + ΔhD) was unable to bind USP7 with comparable potency, whereas ΔhA (removing hairpin A) or ΔhB or ΔhC or ΔhD possessed the ability to bind to USP7 at a similar potency. Taken together, these findings showed a specific interaction between USP7 and hA or hB or hC or hD of the circ‐0100519 (Figure 5D). Similarly, we identified the fragments retaining nt 450−550 of circ‐0100519 bound to NRF2 as efficiently as the entire circ‐0100519, whereas other fragments of circ‐0100519 completely lost the binding capacity, suggesting that nt 450−550 contains the motif that interacts with NRF2. Furthermore, two hairpin structures within nt 450−550 were predicted by RNAfold, which we named hairpin E (hE) and hairpin F (hF). The RNA pull‐down test demonstrated that ΔhE+ ΔhF failed to bind to NRF2, whereas ΔhE or ΔhF was capable of binding to NRF2 (Figure 6E–G). Next, agarose gel electrophoresis analysis and RNA pull‐down assay verified that circ‐0100519‐MUT (1‐160) failed to bind to USP7 and circ‐0100519‐MUT (450‐550) failed to bind to NRF2 (Figure 5H and I). Overexpression of circ‐0100519 notably inhibited the ubiquitination levels of NRF2, however, circ‐0100519‐MUT (450‐550) eliminated this effect (Figure 5J). To identify the domain of NRF2 interacting with circ‐0100519, four truncated NRF2 proteins were generated. Only the nt83‐333 region of NRF2 mutant was found to interact with circ‐0100519 (Figure 5K and L). Therefore, circ‐0100519 interacts with USP7 and NRF2 via distinct motifs containing various hairpin structures to form a circ‐0100519/USP7/NRF2 complex.

### 3.5 NRF2 is a functional downstream mediator of circ‐0100519 and USP7

To further clarify the role of NRF2 in the circ‐0100519 regulation of BC, macrophages (THP‐1 cells) transfected with Vector and NRF2 plasmids were cocultured with exosomes isolated from circ‐0100519 knockdown tumour cells. Colony formation, EdU and transwell assays were used to explore the effects of NRF2 on BC cell proliferation and metastasis in vitro. The results showed that overexpression of NRF2 notably reversed the inhibitory effect of circ‐0100519 knockdown on tumour progression (Figure 6A–C). In addition, to validate the effect of NRF2 in vivo, a macrophage‐depleted diphtheria toxin receptor (DTR) mouse model was constituted. Consistent with the in vitro results, exo‐shcirc‐0100519‐NRF2 therapy increased the tumour size and depraved prognosis compared to the exo‐shcirc‐0100519‐Vector treatment (Figure 6D–F). TUNEL staining indicated fewer TUNEL‐positive cells in the exo‐shcirc‐0100519‐NRF2 group compared to the control (Figure 6G). In contrast to the exo‐shcirc‐0100519‐Vector group, the tumour tissue with the treatment of exo‐shcirc‐0100519‐NRF2 recruited more F4/80^+^CD206^+^ cells (Figure 6H and I). We then investigated the role of NRF2 in BC metastasis by using a lung metastasis model and observed that the tumour burden and incidences of lung metastasis in the exo‐shcirc‐0100519‐NRF2 group were greater than those in the exo‐shcirc‐0100519‐Vector group (Figure 6J and K). We further treated NRF2^M‐KO^ mice with BT‐549 generated exosomes (exosomes were isolated from shNC‐ or shcirc‐0100519‐transfected BT‐549 cells culture supernatants), no significant survival differences were observed between the two groups of mice (Figure 6L and M). Additionally, immunofluorescence studies showed no difference in macrophages polarisation in NRF2^M‐KO^ mice treated with shNC or shcirc‐0100519 (Figure 6N). The proportion of F4/80^+^CD206^+^ cells was noticeably lower in the NRF2 ^M‐KO^ mice group than that in the WT group (Figure S3G). These data suggested that circ‐0100519 promoted BC progression and altered the polarisation of M2‐like macrophages via NRF2 in vivo. This was also confirmed in patient samples, the patient group with higher circ‐0100519 expression had more CD206^+^ cells and greater Ki67 expression (Figure 6O and P).

### 3.6 HIF‐1α serves as an upstream effector to enhance circ‐0100519 transcription

**FIGURE 7:**
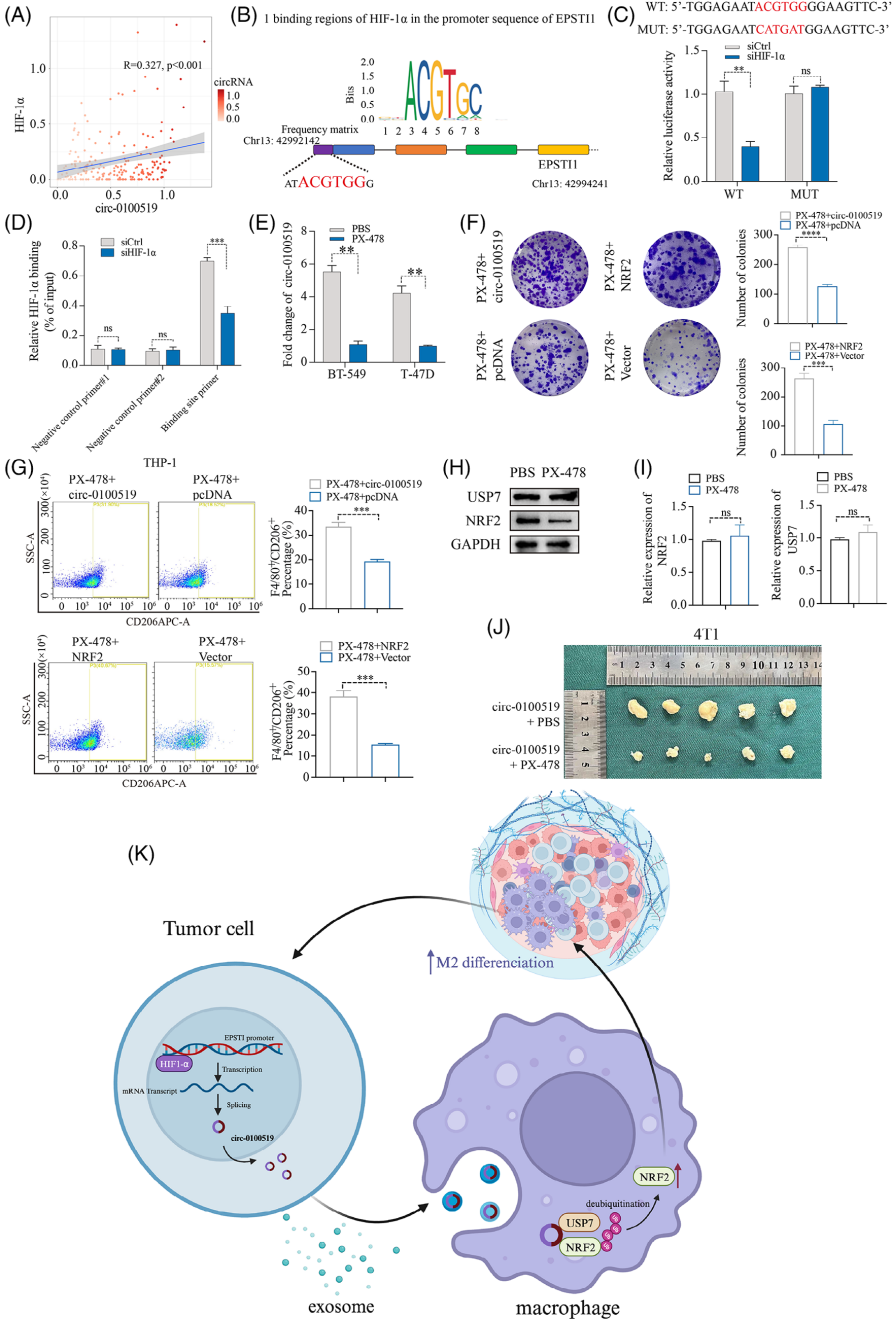
**HIF‐1α serves as an upstream effector to enhance circ‐0100519 transcription**. (A) Relationship between the expression of circ‐0100519 and HIF‐1α according to 196 tissues sequencing. (B) Potential HIF‐1α‐binding site in the genomic region adjacent to the TSS of EPSTI1. (C) Putative or mutant HIF‐1α binding sequences are shown by red characters in the binding areas. Lucifer activity of reporter vectors with the EPSTI1 promoter in BT‐549 cells. (D) HIF‐1α binding to the EPSTI1 promoter in BT‐549 cells was examined using CHIP. Utilised as negative controls were two circ‐0100519 promoter regions that were not anticipated to be bound by HIF‐1α. Data (3 independent biological replicates) are shown as mean ± SD. (E) Relative expression levels of circ‐0100519 after treatment with the HIF‐1α inhibitor PX‐478 assessed by qRT‐PCR. (F) Colony formation assays were used to detect the BC cells' proliferative potential. (G) FCM was used to assess CD206 expression in THP‐1. (F, G: BT‐549 cells were pretreated with 30 µM PX‐478 or PBS for 24 h and meanwhile transfected with circ‐0100519 or pcDNA, and then cocultured with THP‐1 cells. BT‐549 cells were pretreated with 30 µM PX‐478 or PBS for 24 h and cocultured with THP‐1 cells transfected with NRF2 plasmid or Vector) (H, I) Western blotting and qRT‐PCR in THP‐1 cells after pretreated BT‐549 cells with 30 µM PX‐478 or PBS for 24 h and cocultured with THP‐1 cells. (J) Photographs of tumours collected from 4T1‐bearing Balb/c mice with PX‐478 treatment. A representative data set is displayed as mean ± SEM values of three to five independent replicates. ns, not significant, *p < .05, **p < .01, ***p < .001, ****p < .0001.

Based on UCSC (http://genome.ucsc.edu/) and Jasper database (https://jaspar.elixir.no), we found that HIF‐1α might be the potential transcription factor for EPSTI1. Additionally, bioinformatic analysis of 196 tissues sequencing revealed a positive relationship between the expression of circ‐0100519 and HIF‐1α, providing proof for HIF‐1α induced circ‐0100519 upregulation (*R* = 0.327, *p* < .001, Figure 7A). Putative HIF‐1α‐binding sites in the EPSTI1 promoter were identified using NCBI (https://www.ncbi.nlm.nih.gov/mesh/) and Jaspar database, and 24 motifs have been predicted (Table S5). We selected the sequence with the best score for validation. The luciferase experiment revealed that site‐directed mutagenesis could abolish the decreased promoter activity in HIF‐1α‐knockdown BT‐549 cells (Figure 7B and C). The HIF‐1α occupancy at the EPSTI1 promoters in BT‐549 cells were further confirmed by ChIP tests (Figure 7D). Taken together, HIF‐1α is a downstream effector of circ‐0100519 and directly binds the EPSTI1 promoter to enhance its mRNA expression. It was also found that circ‐0100519 expression levels were significantly reduced after HIF‐1α inhibitor PX‐478 treatment (Figure 7E). To further verify the effect of PX‐478 on the entire downstream, colony formation and flow cytometry assays were conducted. The result showed that overexpress circ‐0100519 or NRF2 can rescue the inhibitory effect of PX‐478 on the proliferation of BC cells and M2 macrophages polarisation (Figure 7F and G). We pretreated BT‐549 cells with 30 µM PX‐478 for 24 h and cocultured with THP‐1 cells. The Western blot results showed that PX‐478 had no effect on the protein levels of USP7 in THP‐1 cells, while NRF2 protein levels were inhibited. The qRT‐PCR results verified that PX‐478 had no impact on the mRNA levels of USP7 or NRF2 in THP‐1 cells, as was the case with shcirc‐1000519 transfection in tumour cells (Figure 7H and I). In sum, these data suggested that PX‐478 regulated the downstream pathway of circ‐0100519 by inhibiting HIF‐1α. As predicted, compared to the circ‐0100519 overexpression group, PX‐478 infusions increased the survival time and inhibited the growth and weight of tumours (Figure 7J). Meanwhile, we used GEO (https://www.ncbi.nlm.nih.gov/mesh/) and TCGA database (https://www.cancer.gov/ccg/research/genome‐sequencing/tcga) for external validation and discovered that HIF‐1α and NRF2 were BC tumour promoter which is consistent with our study (Figure S3H and I).

## 4 DISCUSSION

Breast cancer is the leading cause of cancer‐related death in women.^34^, ^35^ Compared with other cancer types, BC has a higher cure rate and a better overall prognosis. For certain individuals, however, recurrence or metastasis are inevitable. As many studies have discovered that circRNAs are crucial for both BC initiation and progression,^36^, ^37^, ^38^, ^39^ we linked circ‐0100519 and secretion of exosomes to the alteration of the tumour microenvironment in this study. Additionally, we clarified the communication between BC cells and macrophages, and the underlying mechanisms of exosomal circ‐0100519 derived from tumour cells on BC progression.

Using high‐throughput sequencing and bioinformatics analysis, we found that circ‐0100519, which is derived from EPSTI1, was notably upregulated in BC tissues and significantly related to macrophages. Rao et al. discovered that EPSTI1 promoted colon cancer progression through the control of N‐cadherin overexpression and E‐cadherin downregulation.^40^ EPSTI1 has also been shown to be essential for ovarian cancer and breast cancer.^41^, ^42^ Based on multivariate cox regression analysis, circ‐0100519 is an independent factor affecting the prognosis of BC patients. Although age and IHC subtype had no statistical significance, these two factors were also important causes for poor prognosis according to the results (median of HR > 1), which is consistent with previous studies.^43^, ^44^ Notably, our results implied a positively correlation between the expression of circ‐0100519 in serum exosomes and corresponding BC tumours, while circ‐0100519 almost undetectable in healthy individuals. This revealed that circ‐0100519 in serum exosomes may serve as a diagnostic biomarker for early BC patients.

Functionally, the exosomes containing circ‐0100519, secreted from cancer cells, can be engulfed by macrophages, thereby influencing the progression of BC by regulating M2‐like macrophage polarisation. TAMs mainly exhibit the M2 phenotype are associated with the poor prognosis of malignant tumour patients.^45^, ^46^ Therefore, targeting M2‐TAMs is a promising therapeutic strategy to changeover the immunosuppressive TME. We offered the initial evidence that exosomal circ‐0100519 may strongly promote the development and metastasis of BC in vitro and in vivo through tumour‐infiltrating macrophage.

Ubiquitination, as a process that is reversible can be eliminated by deubiquitination enzymes (DUBs) and plays an important role in a multitude of cellular activities.^47^ USP7, one of the DUBs, has garnered more and more attention on its role in the initiation and advancement of tumours. For instance, USP7 interacted with TAZ and removed the K48‐linked ubiquitin chains of TAZ to accelerate the progression of head‐neck squamous cell carcinoma.^48^ Dai et al. found that USP7 was crucial to TAM reprogramming in lung cancer, thereby providing new targets for lung cancer clinical therapy.^49^ Additionally, USP7 could regulate lung adenocarcinoma through USP7/Raf‐1/ERK1/2 axis.^50^ The biological function and underlying mechanisms of USP7 in BC, however, need further exploration. NRF2 emerges as an essential transcription factor, integral to both oxidative stress responses and antioxidative fortitude of cells.^51^ Research has revealed that NRF2 is essential for the growth of BC and it may polarise macrophages into M2 phenotype.^52^, ^53^ Our results implied that BC cells secreted exosomes containing circ‐0100519 to macrophages, after which circ‐0100519 can bind with USP7 and NRF2 in macrophages. Moreover, we first determined that circ‐0100519 may work as a scaffold to promote the deubiquitinating effect of USP7 on NRF2, and increased the latter protein level. As we mentioned before, increased NRF2 protein levels promoted M2 macrophage polarisation, which ultimately encouraged the development of BC. We discovered that circRNA can regulate the ubiquitination of NRF2 protein, which is different from the traditional function of circRNAs.

HIF‐1α is a critical transcriptional regulator mediating cellular responses to hypoxia, implicated in the pathogenesis and angiogenesis of tumours, and extensively involved in the modulation of both innate and adaptive immune responses.^54^, ^55^ Existing research indicated that HIF‐1α is overexpressed in BC tissues and correlated with an increased risk of poor prognosis.^56^, ^57^ Song et al. confirms that in BC‐associated macrophages, long noncoding RNAs are regulated by the transcription factor HIF‐1α, playing a vital role in the glycolysis and drug resistance of tumour cells.^58^ Interestingly, 196 BC samples revealed a positive correlation between the expressions of HIF‐1α and circ‐0100519. Furthermore, HIF‐1α was identified as a vital transcription factor of EPSTI1 in our study. PX‐478, a HIF‐1α inhibitor, has been shown to inhibit the protein expression level and transcriptional activity of HIF‐1α, thereby exhibiting effective antitumour activity in various tumours, including nonsmall cell lung cancer, glioma and pancreatic cancer.^59^, ^60^, ^61^ Therefore, it may have applications in tumour therapy. However, there have been few studies on PX‐478 in BC. After applying PX‐478, a significant decrease in the expression of circ‐0100519 was observed. We speculated that PX‐478 may reduce the expression level of circ‐0100519 by inhibiting the transcriptional activity of HIF‐1α. Also, the circ‐0100519‐overexpressed Balb/c mice treated with PX‐478 showed the rescue of tumour progression. These findings suggested the potential of HIF‐1α inhibitor PX‐478 as a novel therapeutic agent to BC and lay the foundation for the subsequent clinical transformation.

In summary, we have identified that circ‐0100519 was significantly expressed in BC and entered TAMs through exosomes. It regulated M2 macrophage polarisation through the circ‐0100519/USP7/NRF2 axis, thereby promoting the progression of BC. We further verified that HIF‐1α may serve as an upstream effector for circ‐0100519. These findings not only reveal the important role of circ‐0100519 in BC development but also provide a therapeutic target for BC, offering new directions for future treatment strategies. However, some limitations exist in the study. We could not exclude the function of circ‐0100519 itself in macrophages. Furthermore, translation of PX‐478 impact on human BC was only assessed constructing a mouse model. To completely evaluate the relevance of our results to human health, more researches need to be investigated to assess the effect of PX‐478 on BC. Also, we did not construct circ‐0100519 RNA antibodies and vaccines to verify its clinical translational potential. Future experimental work focus on this part will be interesting. Our findings thus demonstrate a model mechanism in which the circ‐0100519/USP7/NRF2 axis promotes macrophage polarisation and BC progression. This study provides the knowledge of the crosstalk potential of exosomes and highlighted the promising application of HIF‐1α inhibitor in breast cancer.

## AUTHOR CONTRIBUTIONS

All authors meet authorship requirements. Conception and design were performed by YHZ and XAL. Acquisition, analysis and interpretation of data were taken part by MYZ, XQZ and JJ. Writing, review and/or revision of the manuscript were performed by HFZ and LS. All authors read and approved the final manuscript.

## CONFLICT OF INTEREST STATEMENT

The authors declare that they have no known competing financial interests or personal relationships that could have appeared to influence the work reported in this paper.

## ETHICS STATEMENT

The Nanjing Medical University (NJMU) Institutional Animal Care and Use Committee's regulations were followed in all animal experimentation. Approval of this research was obtained by the Ethics Committee of the First Affiliated Hospital of Nanjing Medical University. All patients who were collected samples had written informed consent by themselves or their relatives.

## Supporting information

FIGURE S1
(A) Heatmap illustrating the circRNA expression variations in 196 tissues from BC patients. (B) Dot plot showing the relative expression of circRNA across several immune cells. A total of 16 circRNAs most strongly associated with each subtype of immune cells were selected (with statistical difference; |*R* ≥ 0.6|). (C–E) FCM was used to assess CD206 or CD86 expression in THP‐1. (BT‐549 cells were treated with shNC or shcircNFATC2_001; shNC or shcirc MCTP1_028; shNC or shcirc‐0100519 before coculturing with preactivated THP‐1.) (F) Risk factors associated with poor prognosis of 60 BC patients were assessed by multivariate cox regression analysis. (G) By employing PCR analysis, the divergent primers for circ‐0100519 could be amplified from cDNA rather than gDNA. The triangles in the figure meant convergent and divergent primers (the two triangles with opposite points referred to convergent primers, the two triangles with opposite bases referred to divergent primers). (H) Circ‐010519 and EPSTI1 mRNAs expression following RNase treatment. (I) RNA abundance of circ‐010519 and EPSTI1 after treatment with Actinomycin. (J) Relative expression levels of circ‐0100519 in subcellular fractions. (K) FISH assays were used to display the expression of circ‐0100519 in BT‐549 (Red). Scale bar = 50 µm. A representative data set is displayed as mean ± SEM values of three or more independent replicates. ns, not significant, *p < .05, **p < .01, ***p < .001, ****p < .0001.

FIGURE S2
(A) The expression levels of circ‐0100519 and EPSTI1 in BT‐549 were assessed by qRT‐PCR. (B, C) FCM was used to assess CD206 expression in BMDM. (BT‐549 cells were treated with shNC or shcirc‐0100519 before coculturing with BMDM.) (D, E) BT‐549 or T‐47D cells were treated with shNC or shcirc‐0100519 before coculturing with THP‐1 D. ELISA was used to identify secreted IL‐6, TNF and MCP‐1 in the supernatants of THP‐1. (E) CCK‐8 assays were used to assess the viabilities of BC cells (the coculture system was treated with the exosome inhibitor GW4869). (F) CCK‐8 assays were used to assess the viabilities of BC cells. (Instead of coculture system, GW4869 or DMSO was directly applied to breast cancer cells.) (G) THP‐1 cells treated with gradient concentrations of exosomes isolated from BT‐549 cells. The relative expression of circ‐0100519 in macrophages was evaluated by qRT‐PCR. (H) qRT‐PCR was used to verify the expression levels of circ‐0100519 in metastatic tumour tissues and nonmetastatic tumour tissues. A representative data set is displayed as mean ± SEM values of three independent replicates. ns, not significant, *p < .05, **p < .01, ***p < .001, ****p < .0001.

FIGURE S3
(A, B) IP/MS analysis showed that USP7 and NRF2 might be the RNA‐binding proteins of circ‐0100519. (C) Colocalisation of circ‐0100519 (RED) with USP7 proteins (GREEN) and NRF2 proteins (PURPLE). Scale bar = 5 µm. (D) Exogenous protein interactions were identified in THP‐1 cells. IgG was used as control. (E) Relative expression levels of circ‐0100519 were assessed by qRT‐PCR in THP‐1 transfected with circ‐0100519 or shcirc‐0100519. (F) The expression of USP7, MDM2 and P53 in protein level after overexpression of circ‐0100519 in THP‐1. (G) FCM was utilised to evaluate CD206 expression in NRF2^M‐KO^ and WT mouse tumours. (H, I) Overall survival of NRF2 low and NRF2 high groups (HIF‐1α low and HIF‐1α high groups) in BC patients was analysed by Kaplan–Meier curves and log‐rank tests. The data were based on GEO and TCGA database. (J) qRT‐PCR was used to simultaneously assess the expression levels of circ‐0100519 or EPSTI1. A representative data set is displayed as mean ± SEM values of three independent replicates. ns, not significant, *p < .05, **p < .01, ***p < .001, ****p < .0001.

Supporting Information

Supporting Information

Supporting Information

Supporting Information

Supporting Information

## Data Availability

The datasets used and/or analysed during the current study are available from the corresponding author on reasonable request. Supplementary Information accompanies this paper as Supplementary Tables and Materials.
