# Supplementary figures and images for: Exosomal circ‐0100519 promotes breast cancer progression via inducing M2 macrophage polarisation by USP7/NRF2 axis
Source: Clin Transl Med. 2024 Aug 6;14(8):e1763. doi: 10.1002/ctm2.1763 (PMC11303452; doi:10.1002/ctm2.1763)

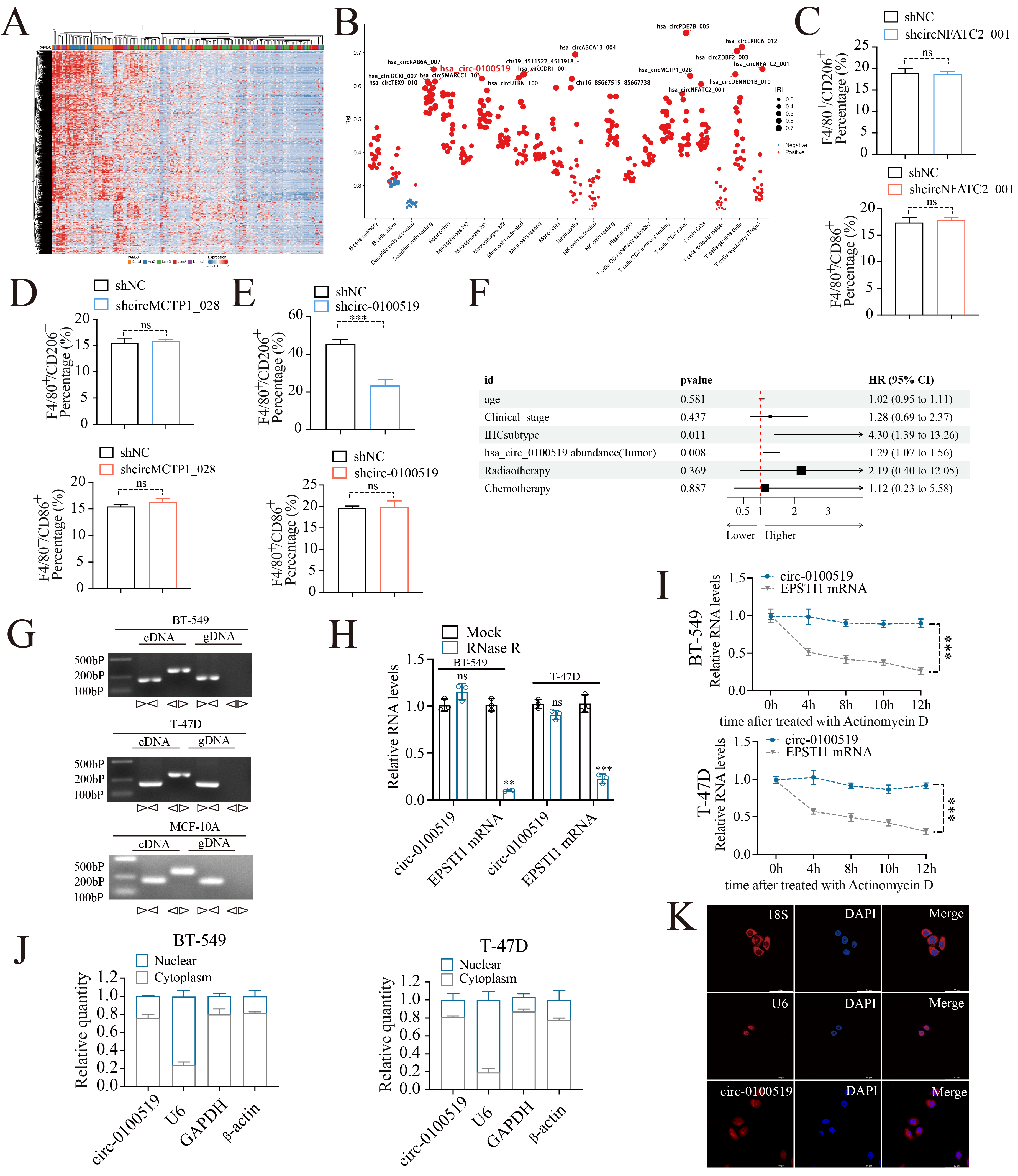

Supplement: Supplementary file 1 — FIGURE S1 (A) Heatmap illustrating the circRNA expression variations in 196 tissues from BC patients. (B) Dot plot showing the relative expression of circRNA across several immune cells. A total of 16 circRNAs most strongly associated with each subtype of immune cells were selected (with statistical difference; |R ≥ 0.6|). (C–E) FCM was used to assess CD206 or CD86 expression in THP‐1. (BT‐549 cells were treated with shNC or shcircNFATC2_001; shNC or shcirc MCTP1_028; shNC or shcirc‐0100519 before coculturing with preactivated THP‐1.) (F) Risk factors associated with poor prognosis of 60 BC patients were assessed by multivariate cox regression analysis. (G) By employing PCR analysis, the divergent primers for circ‐0100519 could be amplified from cDNA rather than gDNA. The triangles in the figure meant convergent and divergent primers (the two triangles with opposite points referred to convergent primers, the two triangles with opposite bases referred to divergent primers). (H) Circ‐010519 and EPSTI1 mRNAs expression following RNase treatment. (I) RNA abundance of circ‐010519 and EPSTI1 after treatment with Actinomycin. (J) Relative expression levels of circ‐0100519 in subcellular fractions. (K) FISH assays were used to display the expression of circ‐0100519 in BT‐549 (Red). Scale bar = 50 µm. A representative data set is displayed as mean ± SEM values of three or more independent replicates. ns, not significant, *p < .05, **p < .01, ***p < .001, ****p < .0001. [file CTM2-14-e1763-s003.png]

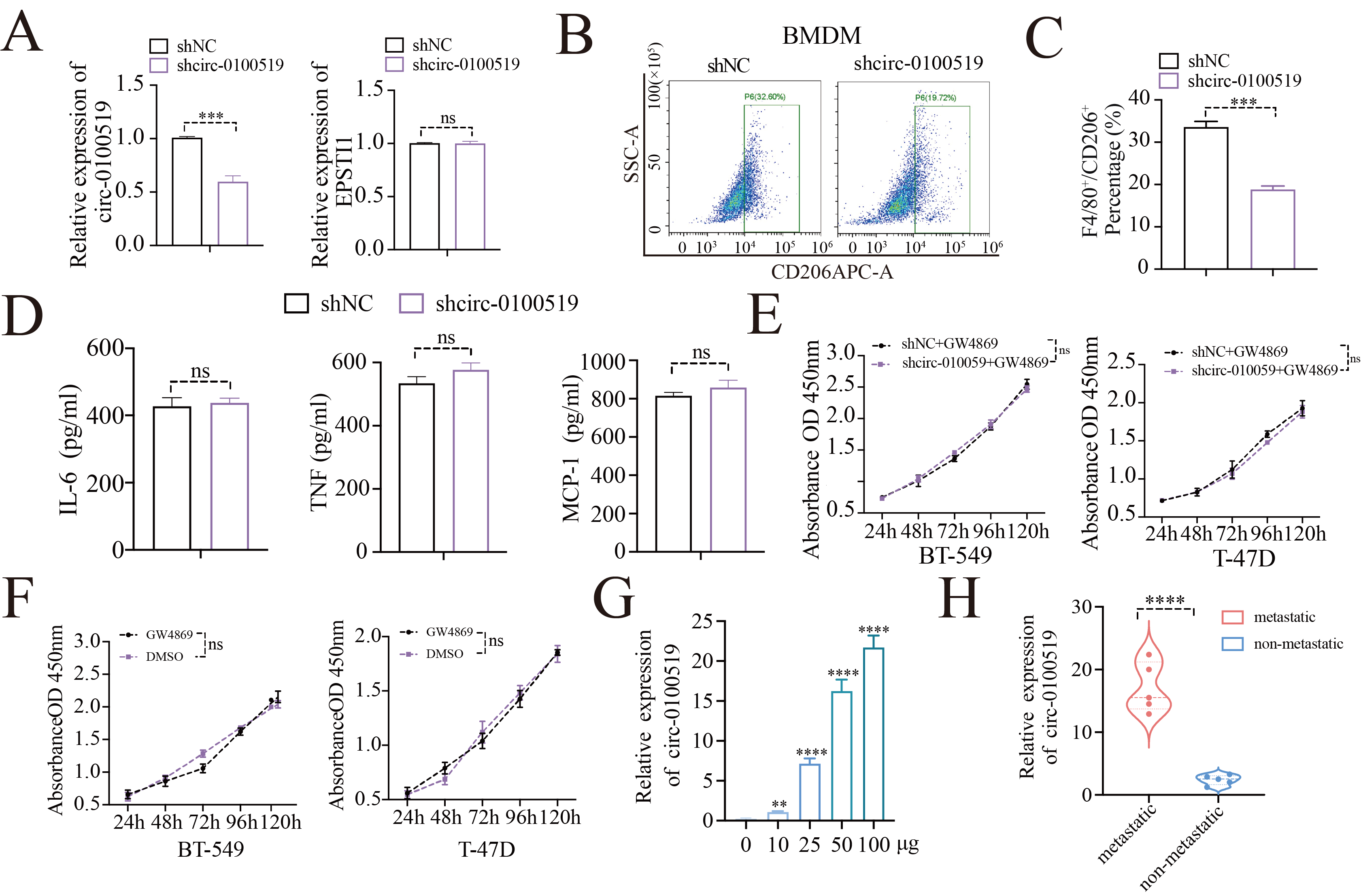

Supplement: Supplementary file 2 — FIGURE S2 (A) The expression levels of circ‐0100519 and EPSTI1 in BT‐549 were assessed by qRT‐PCR. (B, C) FCM was used to assess CD206 expression in BMDM. (BT‐549 cells were treated with shNC or shcirc‐0100519 before coculturing with BMDM.) (D, E) BT‐549 or T‐47D cells were treated with shNC or shcirc‐0100519 before coculturing with THP‐1 D. ELISA was used to identify secreted IL‐6, TNF and MCP‐1 in the supernatants of THP‐1. (E) CCK‐8 assays were used to assess the viabilities of BC cells (the coculture system was treated with the exosome inhibitor GW4869). (F) CCK‐8 assays were used to assess the viabilities of BC cells. (Instead of coculture system, GW4869 or DMSO was directly applied to breast cancer cells.) (G) THP‐1 cells treated with gradient concentrations of exosomes isolated from BT‐549 cells. The relative expression of circ‐0100519 in macrophages was evaluated by qRT‐PCR. (H) qRT‐PCR was used to verify the expression levels of circ‐0100519 in metastatic tumour tissues and nonmetastatic tumour tissues. A representative data set is displayed as mean ± SEM values of three independent replicates. ns, not significant, *p < .05, **p < .01, ***p < .001, ****p < .0001. [file CTM2-14-e1763-s002.png]

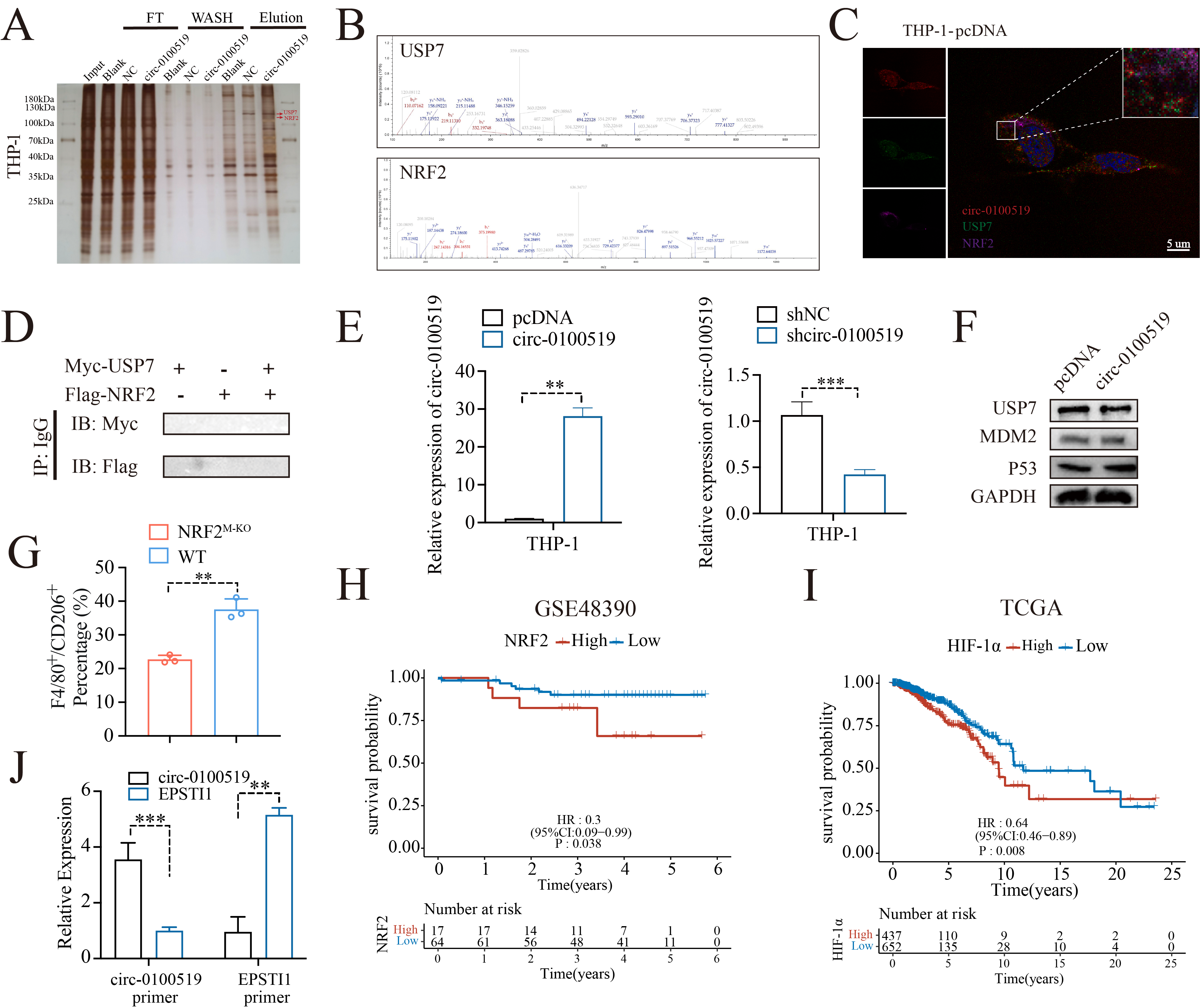

Supplement: Supplementary file 3 — FIGURE S3 (A, B) IP/MS analysis showed that USP7 and NRF2 might be the RNA‐binding proteins of circ‐0100519. (C) Colocalisation of circ‐0100519 (RED) with USP7 proteins (GREEN) and NRF2 proteins (PURPLE). Scale bar = 5 µm. (D) Exogenous protein interactions were identified in THP‐1 cells. IgG was used as control. (E) Relative expression levels of circ‐0100519 were assessed by qRT‐PCR in THP‐1 transfected with circ‐0100519 or shcirc‐0100519. (F) The expression of USP7, MDM2 and P53 in protein level after overexpression of circ‐0100519 in THP‐1. (G) FCM was utilised to evaluate CD206 expression in NRF2M‐KO and WT mouse tumours. (H, I) Overall survival of NRF2 low and NRF2 high groups (HIF‐1α low and HIF‐1α high groups) in BC patients was analysed by Kaplan–Meier curves and log‐rank tests. The data were based on GEO and TCGA database. (J) qRT‐PCR was used to simultaneously assess the expression levels of circ‐0100519 or EPSTI1. A representative data set is displayed as mean ± SEM values of three independent replicates. ns, not significant, *p < .05, **p < .01, ***p < .001, ****p < .0001. [file CTM2-14-e1763-s005.png]
