# Supplementary material for: Exosomal circ‐0100519 promotes breast cancer progression via inducing M2 macrophage polarisation by USP7/NRF2 axis
Source: Clin Transl Med. 2024 Aug 6;14(8):e1763. doi: 10.1002/ctm2.1763 (PMC11303452; doi:10.1002/ctm2.1763)
Supplement: Supplementary file 6 — Supporting Information [file CTM2-14-e1763-s001.doc]

**Supplementary Table 3. Primers and si/sh Target sequences used for Quantitative RT-PCR or transfection**

| RNA | 5'to 3' |
| --- | --- |
| circ-0100519  (Convergent) | F: ATCCTCAACATGGAAACTTCCTATC  R: GCTCCGCAATTCTTTGTATCTC |
| circ-0100519  (Divergent) | F: TGTCAAAGTGCTGTTTGTGGC  R: TTAGCTCTGTTCTGCTCCTTCC |
| EPSTI1 | F: ACCCGCAATAGAGTGGTGAAC  R: GCTATCAAGGTGTATGCACTTGT |
| β-actin | F: CATGTACGTTGCTATCCAGGC  R: CTCCTTAATGTCACGCACGAT |
| U6 | F: CTCGCTTCGGCAGCACA  R: AACGCTTCACGAATTTGCGT |
| USP7 | F: GATGAAAAGTCGTTCAGTCGTCG  R: TTTGAATCCCACGCAACTCCA |
| NRF2 | F: ACACGGTCCACAGCTCATC  R: TCTTGCCTCCAAAGTATGTCAA |
| GAPDH | F: TGTCAAGCTCATTTCCTGGTAT  R: CTCTCTTCCTCTTGTGCTCTTG |
| HIF-1α/EPSTI1  binding site | F: CACCCTCCTCCAAGTGGTTC  R: GGGTCACAATGCCGTCTACA |
| HIF-1α/EPSTI1 negative control#1 | F: GCACACTGTTCTGCACCGTT  R: CACTGAGGCCCCGTATTCTC |
| HIF-1α/EPSTI1  negative control#2 | F: AGTCCAGCTCCTAGGAAGAC  R: GTGTGCAACGGTGCAGAACA |
| shcirc-0100519 | AACAUCAAAAGCACAAGUGCA |
| shNC | CCGCCCUAAGGUUAACUCGCUC |
| siHIF-1α | GGGATTAACTCAGTTTGAACT |
